# Development and validation of cuproptosis-related lncRNAs associated with pancreatic cancer immune microenvironment based on single-cell

**DOI:** 10.3389/fimmu.2023.1220760

**Published:** 2023-09-25

**Authors:** Yimeng Sun, Lin Yao, Changfeng Man, Zhenjun Gao, Rong He, Yu Fan

**Affiliations:** ^1^ Cancer Institute, Affiliated People’s Hospital of Jiangsu University, Zhenjiang, Jiangsu, China; ^2^ Department of Gastroenterology, Qingpu Branch of Zhongshan Hospital Affiliated to Fudan University, Shanghai, China

**Keywords:** cuproptosis, single-cell sequence, immunotherapy, tumor immune microenvironment, pancreatic cancer, prognostic signature

## Abstract

**Background:**

Cuproptosis, a novel mode of cell death associated with the tricarboxylic acid (TCA) cycle, is relevant to the development of cancer. However, the impact of single-cell-based Cuproptosis-associated lncRNAs on the Tumor immune microenvironment (TIME) of Pancreatic adenocarcinoma (PAAD) and its potential value for individualized immunotherapy has not been clarified.

**Methods:**

14 immune-related CRGs were screened by exploring the interaction between differentially expressed Immune-Related Genes (IRGs) and Cuproptosis-Related Genes (CRGs) in PAAD. Next, the expression amount and expression distribution of CRGs in single-cell samples were analyzed by focusing on 7-CRGs with significant expressions. On the one hand, MAP2K2, SOD1, and VEGFA, which were significantly differentially expressed between PAAD sites and normal tissues adjacent to them, were subjected to immunohistochemical validation and immune landscape analysis. On the other hand, from these 7-CRGs, prognostic signatures of lncRNAs were established by co-expression and LASSO-COX regression analysis, and their prognostic value and immune relevance were assessed. In addition, this study not only validated the hub CRGs and the lncRNAs constituting the signature in a PAAD animal model treated with immunotherapy-based combination therapy using immunohistochemistry and qRT-PCR but also explored the potential value of the combination of targeted, chemotherapy and immunotherapy.

**Results:**

Based on the screening of 7-CRGs significantly expressed in a PAAD single-cell cohort and their co-expressed Cuproptosis-Related lncRNAs (CRIs), this study constructed a prognostic signature of 4-CRIs named CIR-score. A Nomogram integrating the CIR-score and clinical risk factors was constructed on this basis to predict the individualized survival of patients. Moreover, high and low-risk groups classified according to the median of signatures exhibited significant differences in clinical prognosis, immune landscape, bioenrichment, tumor burden, and drug sensitivity. And the immunohistochemical and qRT-PCR results of different mouse PAAD treatment strategies were consistent with the trend of inter-group variability in drug sensitivity of hub CRGs and CIR-score. The combination of immunotherapy, targeted therapy, and chemotherapy exhibited a better tumor suppression effect.

**Conclusion:**

CIR-score, as a Cuproptosis-related TIME-specific prognostic signature based on PAAD single cells, not only predicts the prognosis and immune landscape of PAAD patients but also provides a new strategy for individualized immunotherapy-based combination therapy.

## 1 Introduction

Worldwide, PAAD ranks seventh in cancer-related deaths (1). As a highly fatal disease, PAAD mortality is expected to continue to rise in the coming decades, with more than 800,000 deaths expected by 2040 (2). A major feature of human PAAD is the lack of effective anti-tumor immunity. Multiple immunosuppressive and evasion mechanisms, established from the early steps of tumor transformation, effectively protect PAAD from immunity (3). Suppressive TIME, consisting of multiple immune cell populations and stromal components in tumor islets, is not only a fertile ground for PAAD development, progression, and metastasis but also a great obstacle to immunotherapy (4). Single-cell sequencing, as a technique for analysis at the individual cellular level, overcomes the problem of cellular heterogeneity (5). It not only better reflects how the different cell populations that makeup TIME regulate different tumor states from a more microscopic perspective, but also has important implications for unraveling the mechanisms of cancer progression and immunotherapeutic response (6, 7).

Cuproptosis refers to a novel form of cell death in the TCA cycle through the binding of copper to lipoacylase leading to subsequent protein aggregation, proteotoxic stress, and ultimately cell death (8, 9).CRGs and lipoacylated proteins abundance are highly correlated in human tumors, and cell lines with high levels of lipoacylated proteins are sensitive to copper-induced cell death (10). Considering the increased demand for copper by cancer cells, this suggests the potential value of copper ion carrier therapy in tumors with this metabolic profile. Previous studies have shown that serum copper ion concentrations are significantly elevated in patients with PAAD (11). Furthermore, in a mouse model of PAAD, not only did tumor growth rates increase significantly in those chronically exposed to elevated copper levels, but the copper chelator TTM delayed angiogenesis in precancerous lesions and the growth of advanced tumors to some extent (12).

lncRNAs are non-coding RNAs greater than 200 nucleotides in length and transcribed by RNA polymerase II. lncRNAs’ aberrant expression of oncogenic or tumor suppressive effects, high tissue and disease specificity, and ability to modulate TIME make them potential candidates for biomarker and cancer vaccine development (13, 14). lncRNAs not only participate in RNA regulatory mechanisms and control the expression of their downstream target genes, but can also mediate a range of multiple cellular processes through chromatin reprogramming, cis- or trans-regulation of neighboring genes, and post-transcriptional regulation of mRNA processing (15). In the current PAAD studies, proliferation and metastasis have been clearly shown to be affected by the negative regulation of the miR-34 transcriptional pathway by lncRNA HOTAIR (16), the negative regulation of HIF-1α pathway by the reduced expression of lncRNA ENST00000480739 (17), the miR-448 sponge pathway participated by lncRNA PVT1 (18), the inhibition of the oncogene E-cadherin pathway by lncRNA MALAT1 (19), and the regulation of the HOX gene pathway by lncRNA HOTTIP (20). Apart from affecting PAAD proliferation and metastasis, lncRNAs can also regulate TIME via macrophages (21), PD-1 on the surface of T cells (22), etc. In the field of PAAD, Chen H et al. have conducted some studies on the prognostic value of Cuproptosis-associated lncRNAs (23), but there is still a gap in the exploration of Cuproptosis-associated lncRNAs and their characteristic TIME based on PAAD single-cell data.

Given the limited evidence for effective early detection and therapeutic interventions for PAAD, a unified predictive approach needs to be incorporated into strategies for early detection and guiding treatment. In this study, CRGs with immune-related potential prognostic value were screened using differential expression levels and the degree of interaction with IRGs as indicators. The immune landscape of high- and low-expression cohorts of these genes were analyzed on the one hand, and the differences in expression and distribution of these genes in different cell clusters were explored individually at the single-cell level on the other. Subsequently, a prognostic signature of 4-CRIs named CIR-score was constructed based on the 9 most prognostic CRIs screened. This new Cuproptosis-related prognostic signature not merely reflects the tumor load, immune landscape, and immune checkpoint sensitivity of PAAD but also well predicts patient survival and drug sensitivity. Finally, a cohort of four PAAD mouse models with different treatment regimens was used to validate hub lncRNAs of signature and differentially expressed CRGs with prognostic value. The promising tumor suppressive effect demonstrated by immunotherapy combined with targeted therapy and chemotherapy also provides innovative therapeutic options for PAAD.

## 2 Materials and methods

### 2.1 Data acquisition and processing

**Figure 1:**
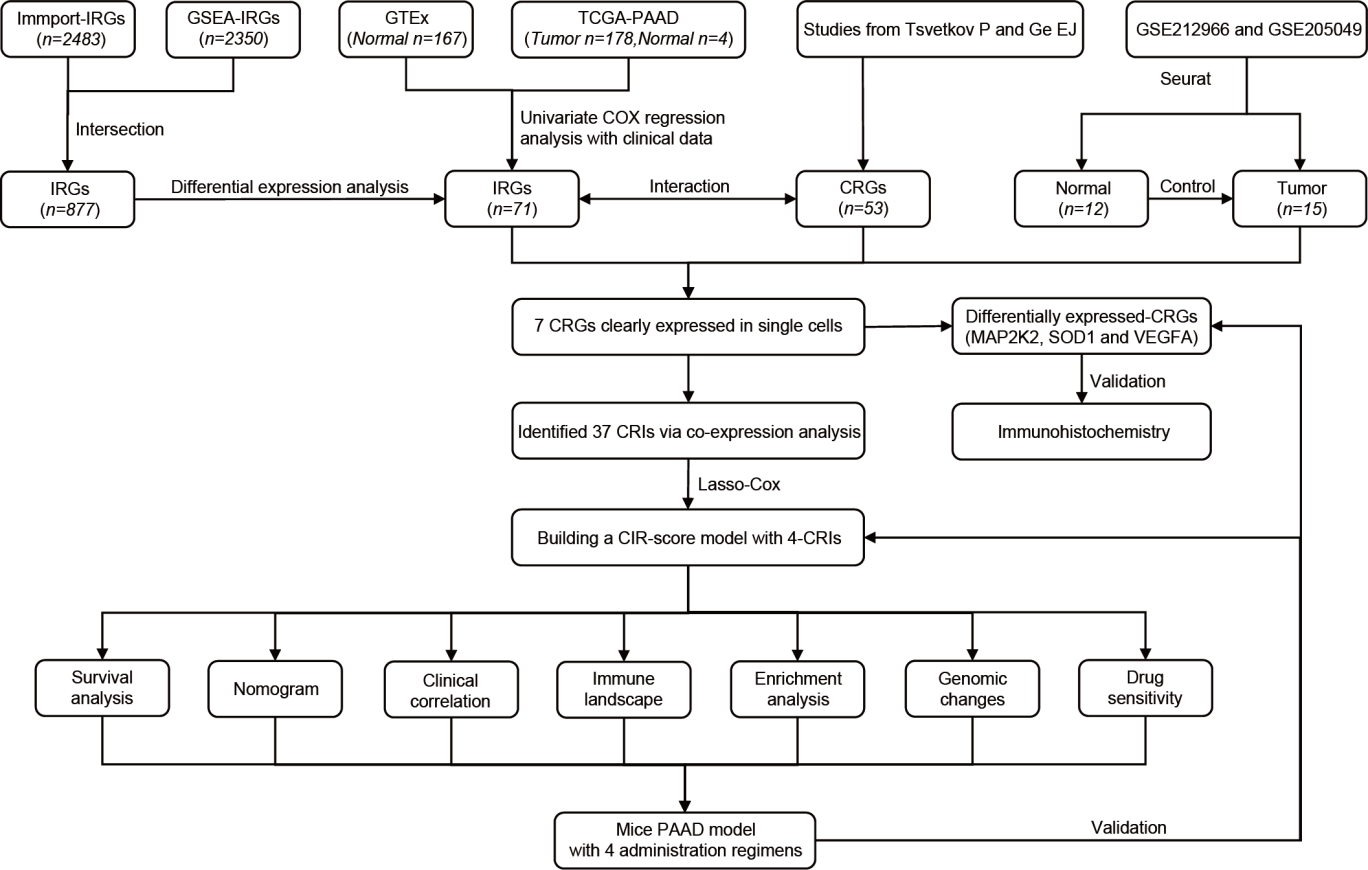
The flowchart of our study process.

The overall flow of this study is shown in the figure (**Figure 1**). A total of 53 CRGs were summarized from previous studies (10, 24). This method of summarizing CRGs has been used by Cai Z et al. in the field of renal clear cell carcinoma (25). A total of 2350 IRGs were screened from the Gene Set Enrichment Analysis database (GSEA, https://www.gsea-msigdb.org/gsea/index.jsp) based on 10 tumor immune-related datasets. A total of 2483 IRGs were downloaded from the Immport database (Updated. July 2020, https://www.immport.org). 178 PAAD samples and 4 normal tissue samples were obtained from The Cancer Genome Atlas Program (TCGA, https://portal.gdc.cancer.gov/). The samples were collated for quantitative gene expression data and clinical data. Transcriptomic data of 167 normal pancreatic tissues were obtained from the Genotype-Tissue Expression Project database (GTEx, https://gtexportal.org/). The cohorts of TCGA-PAAD and GTEx were matched. Single-cell data were obtained from GSE212966 and GSE205049 in the Gene Expression Omnibus database (GEO, https://www.ncbi.nlm.nih.gov/geo/).

### 2.2 Screening and interaction analysis of CRGs and IRGs

The 877 IRGs obtained by taking the intersection of two databases, GSEA and Immport, were subjected to analysis of variation and Univariate COX in the TCGA-PAAD cohort (p=0.05). 71 differentially expressed IRGs with prognostic values were obtained. The String database (https://cn.string-db.org) and Cytoscape software (version 3.9.0) were used to explore the interaction between the two groups of 53 CRGs and 71 IRGs, and only genes with intergroup interactions were retained. Immunohistochemical results of differentially expressed CRGs were obtained through the Human Protein Atlas database (HPA, https://www.proteinatlas.org/).

### 2.3 Immune landscape of differentially expressed CRGs

The TIME evaluation (StromalScore, ImmuneScore, and ESTIMATEScore) of the three immune-related CRGs with significant differential expression was evaluated using the “ESTIMATE” package. “CIBERSORT” was used to obtain the results of immune cell infiltration in different groups with high and low expression of CRGs. The correlation between different immune cells and the expression of different CRGs was obtained by Spearman correlation analysis, and the correlation coefficients and p-values were calculated. The “ggplot2” package was used to draw a Lollipop plot of the correlation between genes and immune cells. The “limma” package and the “corrplot” package were used to plot the correlation heat map between different CRGs and immune checkpoints.

### 2.4 Expression and distribution of CRGs in single cells

Single-cell RNA sequencing (scRNA-seq) count matrices for all 6 PAAD samples were downloaded from GSE212966. These 6 matrices were combined into 1 Seurat object using the CreateSeuratObject function (“Seurat” R package, version 4.3.0). Single cells with more than 2500 or less than 200 genes detected per cell or with a percentage of mitochondria-derived UMI counts greater than 5% were considered low-quality cells and filtered out for further analysis. Single-cell data from 6 PAAD samples were normalized on the basis of the first 2000 high-variable genes (HVG). Subsequently, similar cells were identified using the FindNeighbors function in the “Seurat” package. The FindClusters function (resolution=0.4) was used to identify the major cell clusters. Based on this, UMAP non-linear dimensionality reduction is performed on these cell clusters. The results of the dimensionality reduction were visualized using DimPlot. Cell clusters were identified using the “SingleR” package, and annotation of cell clusters was done based on marker genes obtained from previous studies. Finally, the accuracy of the annotation is verified using the plotScoreHeatmap function. Using the FeaturePlot function, the expression and distribution of the target CRGs are marked on the UMAP map. In addition, GSM6567169 and GSM6567170 with incomplete data and GSM6567167 with too low quality were deleted, and the same analysis as above was done for 3 single cell samples (GSM6567165, GSM6567166, and GSM6567171) with normal tissues adjacent to PAAD from GSE212966 as controls. Furthermore, single-cell sequencing data from the 9-case PAAD samples and their neighboring 9-case normal tissue samples of GSE205049 were included in the study. They were combined into one Seurat object for cancer tissues and one Seurat object for normal tissues using the same data analysis methods described above. The GSE205049 dataset was used as a complement and support to the results of the analysis of the top CRGs based on the samples from GSE212966.

### 2.5 Identification of Cuproptosis-associated lncRNAs signature with prognostic significance in PAAD

7 CRGs that were both co-expressed with IRGs and significantly expressed in PAAD single-cell data were screened. Wilcoxon test was used to obtain lncRNAs with co-expression relationships with these 7 CRGs (correlation coefficient >0.4, p<0.001). “ggalluvial” and “ggplot2” packages were used to draw a correlation Sankey diagram. Next, to further filtrate prognosis-related lncRNAs, we collected 9 CRIs by univariate Cox regression analysis (p< 0.05). Least absolute shrinkage and selection operator (LASSO) regression analysis and lambda spectra were used to screen prognosis-associated lncRNAs to prevent overfitting when constructing prognostic risk models. The area under the ROC curve (AUC) was calculated for these models. When the AUC value reached the maximum value, the model was indicated as the best candidate. Based on the 4 CRIs identified, a new PAAD risk scoring model was identified. This model was named The Cuproptosis-related Immune Risk Score (CIR-score) in this study, and the risk score for each PAAD patient can be derived using the following equation (β: coefficients, Exp: gene expression level):.


$$CIRscore=\Sigma (\beta_{i}\times Exp_{i})$$

In addition, to demonstrate the superiority of the signature in distinguishing between high and low-risk groups of patients, the “scatterplot3d” package was used to perform a principal component analysis (PCA) to visualize the distribution pattern of the CIR-score in the PAAD sample.

### 2.6 Independent prognostic and clinical correlation analysis

The CIR-score was analyzed by univariate and multifactorial Cox regression. The 1-, 3-, and 5-year ROC curves were plotted using the “timeROC” package. The ROC curves of CIR-score and clinical indicators were obtained in the same way as above. The Concordance Index (C-index) was calculated for the CIR-score and clinical indicators, respectively, to evaluate the predictive power of the signature. The prognosis of individual PAAD patients was evaluated by plotting the CIR-score-related Nomogram with the “regplot” package. The calibration curves of the Nomogram were plotted using the Calibrate function. Whereafter, the TCGA-PAAD cohort were randomly grouped into a train set and a test set. The randomness of the grouping was demonstrated by performing a round-robin for clinical traits and obtaining p values (p>0.05) for the differences in clinical traits between the train and test sets. The “pheatmap” package was used to map the differences in CIR-score expression patterns between the TCGA-PAAD cohort, the training set, and the test set for the high and low-risk groups. “survival” and “survminer” packages were used to compare the survival differences between high and low-risk groups. Overall survival (OS) and progression-free survival (PFS) of PAAD patients were analyzed separately using the Kaplan-Meier (KM) method. Besides, clinical traits were grouped and cycled for each group to compare survival differences between high and low-risk groups with different clinical characteristics.

### 2.7 Immune microenvironment landscape analysis

The “GSEABase” package was used to perform a single sample Gene Set Enrichment Analysis (ssGSEA) of immune-related functions in the high- and low-risk groups. The results of the “CIBERSORT” package were used to compare the differences in the infiltration of all 22 immune cell types in the high and low-risk groups. Spearman correlation analysis was used to calculate correlation coefficients and p-values by the immune cell cycle and to plot the correlation between the 4 immune cells with the most significant differences and the CIR-score. Considering that the TISIDB database (http://cis.hku.hk/TISIDB/) is an integrated repository portal for tumor-immune system interactions. To explore the correlation between CIR-score and immune checkpoints, this study summarized the specific immune checkpoint genes of these four immune cells from the TISIDB database. The “corrplot” package was used to visualize the correlations separately.

### 2.8 Functional analysis and tumor mutation burden analysis

The “limma” package was used to screen the differentially expressed genes (DEGs) in high and low-risk groups (∣log2FC∣>1, FDR<0.05). GO and KEGG analyses of DEGs were performed by enrichGO function and enrichKEGG function. Among them, GO analysis included 3 domains of biological process (BP), cellular component (CC), and molecular function (MF). Next, tumor mutation burden (TMB) values were obtained for each sample based on the mutation data downloaded and collated from the TCGA-PAAD cohort. The TMBs of the high- and low-risk groups were compared, and the differences were visualized as violin plots using the ggviolin function. The samples were divided into high and low TMB groups according to the median, and the survdiff function and ggsurvplot function were used to analyze the difference in OS between the high and low mutation load groups. On this basis, survival curves for TMB combined with patient risk were plotted in the same way to further explore the correlation between CIR-score and TMB. Moreover, considering Tumor Immune Dysfunction and Exclusion (TIDE, http://tide.dfci.harvard.edu/) is a database that allows estimating multiple published transcriptomic biomarkers based on tumor pre-treatment expression profiles to predict patient response. Its scoring of PAAD samples was used to analyze differences in potential immune escape potential between high and low-risk groups.

### 2.9 Drug sensitivity analysis

Immunotherapy sensitivity scores for the TCGA-PAAD cohort were downloaded from The Cancer Immunome Atlas (TCIA, https://www.tcia.at/home) database. The TCIA database provides comprehensive immune next-generation sequencing data (NGS) from TCGA and other data sources for 20 solid cancer genomic analysis results. The “ggpubr” package was used to compare the immunotherapy sensitivity of different risk groups by combining the expression of CRGs, hub lncRNAs, and CIR-score size for each sample. The “pRophetic” package was used to calculate the difference in Half Maximal Inhibitory Concentration (IC50) of the different drugs between the different groups, the lower the IC50, the more sensitive the patient was to the drug.

### 2.10 Validation of hub CRGs and lncRNAs in the immune, targeted combination chemotherapy mouse PAAD model

#### 2.10.1 Mice and drugs

6-8 week female mice (C57BL/6). Mouse pancreatic cancer PANC-02 cells. Nanoalbumin paclitaxel (Jiangsu Hengrui Pharmaceutical Co., Ltd., Specification: 100 mg, Lot No.: 220310AF), Gemcitabine, Recombinant Human Vascular Endothelial Inhibitor Injection (Shandong Xiangsheng Biopharmaceutical Co., Ltd., Specification: 15 mg/3 ml, Lot No.: 202109053), PD-1 monoclonal antibody (Jiangsu Hengrui Pharmaceutical Co., Ltd., Specification: 200 mg, Lot No. 202009043A). The above drugs were dissolved and diluted with saline to the desired concentration.

#### 2.10.2 PAAD model preparation

The cultured mouse pancreatic cancer cells PANC-02 cell suspension was collected at a concentration of 5×10^7^ cells/ml and inoculated subcutaneously in the right axilla of mice at 0.1 ml each. The mice transplanted tumors were measured with vernier calipers to measure the diameter of the transplanted tumors, and the animals were randomly grouped when the tumors grew to 100 mm³. At the same time, each group of mice started to administer the drug, with 6 mice in each group.

#### 2.10.3 Grouping and drug administration regimen

The experimental groupings in this study were a, b, c and d. Group-a was the model control group; group-b was the chemotherapy group; group-c was the Recombinant human endostatin + immunotherapy group (PD-1 Monoclonal Antibody group); and group-d was the combination of Recombinant human endostatin + immunotherapy (PD-1 Monoclonal Antibody) + chemotherapy group. The model control mice in group-a were administered with saline intravenous injection, 3 times on days 1, 4, and 7 after grouping. The chemotherapy mice in group-b were treated with Nanoalbumin Paclitaxel injection (20 mg/kg) and Gemcitabine (50 mg/kg) for 3 times on days 1, 4, and 7 after grouping. The Recombinant human endostatin + immunotherapy mice in group-c were treated with Recombinant human endostatin (20 mg/kg) for 15 consecutive days and PD-1 Monoclonal Antibody (200 μg/20g) for 3 times on days 1, 4, and 7 after grouping. The Recombinant human endostatin + immunotherapy + chemotherapy mice in group-d were treated with a combination of Recombinant human endostatin (20 mg/kg) for 15 consecutive days, PD-1 Monoclonal Antibody (200 μg/20g) for 3 times on days 1, 4, and 7 after grouping and Nanoalbumin Paclitaxel (20 mg/kg) + Gemcitabine (50 mg/kg) for 3 times on days 1, 4, and 7 after grouping. More detailed grouping and dosing regimen are shown in a table (**Supplementary Table S1**).

#### 2.10.4 *In vivo* imaging

The tumor marker probe HGC675 (BioActs, LOT: AQN00037) was injected into the tail vein at 0W, 1W, and 2W of administration, respectively, 200 μl/each, and fluorescence *in vivo* imaging was performed at the small animal *in vivo* 3D imaging system IVIS^®^ Spectrum (PerkinElmer) 4 h after injection. Excitation wavelength: 635 nm, emission wavelength: 670 nm.

#### 2.10.5 Immunohistochemical detection of hub CRGs

After the tumor tissues of each group of mice were rinsed, fixed, and dehydrated, the tissues were paraffin-embedded and the wax blocks were fixed on a microtome for continuous sectioning (4 μm thick). Routine immunohistochemistry was performed to detect the expression of 3 hub CRGs in PAAD tumor tissues under 4 different dosing regimens. Antibody information: rabbit anti SOD1 (proteintech, 10269-1-AP) at a dilution ratio of 1:500. rabbit anti VEGFA (proteintech, 19003-1-AP) at a dilution of 1:100. Dilution ratio 1:1000 for mouse anti MEK2 (proteintech, 67410-1-Ig). The kit used MaxVision-mouse/rabbit (Fuzhou Maishin Biotechnology Co., KIT-5010). DAB was used for color development, hematoxylin contrast staining, and observation under light microscopy. Image-Pro Plus (version 6.0) was used to analyze the immunohistochemical results.

#### 2.10.6 Tissue detection of hub lncRNAs

Total RNA was extracted from tissues using TRIzol reagent (Invitgen, MA, USA). extracted RNA was subjected to quantitative polymerase chain reaction using the One Step TB Green™ PrimeScript™ RT-PCR Kit II (SYBR Green). qRT-PCR was performed with SYBR Green PCR Master Mix (Vazyme) in a fluorescent quantitative PCR cycler (ABI Step one plus Real time-PCR system, USA). GAPDH was used as an internal control, and the results of each sample were normalized to GAPDH expression. The sequences of the primers are shown in **Supplementary Table S2**.

### 2.11 Statistical analysis

All data analyses were performed using R software (version 4.2.1, http://www.R-project.org) and GraphPad Prism software (version 8.0.2). Where not explicitly mentioned, thresholds for statistical significance were considered as P< 0.05, with P values being bilateral.

## 3 Results

### 3.1 Development of immune-related CRGs

**Figure 2:**
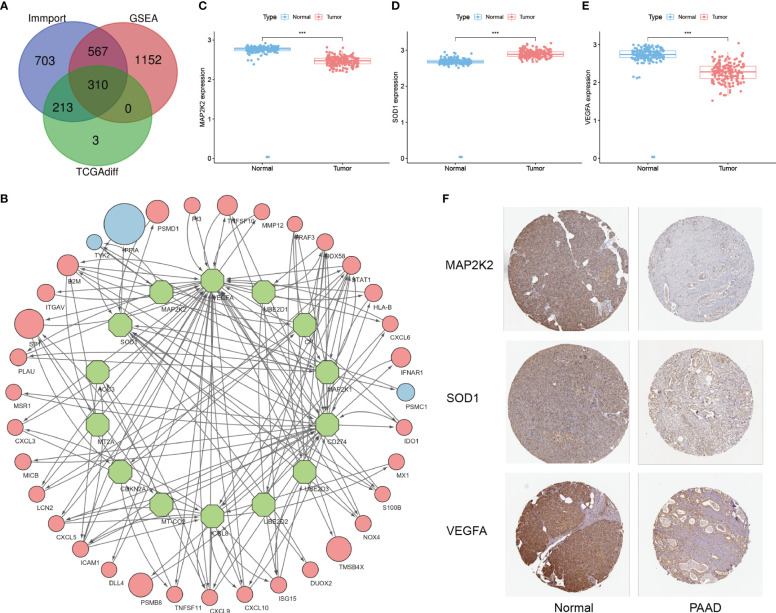
Development of immune-related CRGs. **(A)** Intersection of IRGs from the Immport database, 10 immune-related subsets of IRGs from GSEA and DEGs from the TCGA-PAAD cohort **(B)** 14 CRGs have significant interactions with the 35 IRGs screened (Green nodes: CRGs; Red/Blue nodes: IRGs with high/low expression in PAAD; arrows: direction of action; nodes size: HR value) **(C–E)** Differential expression of MAP2K2, SOD1, and VEGFA in TCGA-PAAD cohort and GTEx cohort ("***": P<0.001) **(F)** Immunohistochemical results.

First, a total of 4833 IRGs were summarized from the Immport database and the 10 GSEA tumor immune-related subsets. 310 of them with significant differences constituted the IRGs cohort by comparing the mRNA expression levels between tumor tissues in the TCGA-PAAD cohort and adjacent normal tissues in the GTEx cohort (**Figure 2A**, **Supplementary Table S3**). Then, 53 CRGs were summarized from the study of Tsvetkov P and Ge EJ, constituting the CRGs cohort (**Supplementary Table S4**). The within-group relationships between the IRGs cohort and the CRGs cohort were eliminated, and only the between-group interaction relationships were retained. The results showed that 14 CRGs interacted significantly with 35 IRGs (**Figure 2B**, **Supplementary Table S5**). Among these 14 immune-related CRGs, MAP2K2, SOD1, and VEGFA were most significantly differentially expressed between the TCGA-PAAD cohort and the GTEx cohort (**Figure 2C-E**). Next, to analyze the reliability of the differences, immunohistochemical results from the HPA database were selected for validation (**Figure 2F**). Among them, the immunohistochemical results of MAP2K2 and VEGFA were consistent with the mRNA differential expression results. MAP2K2 staining in normal pancreatic tissues (Staining: High, Intensity: Strong, Quantity:>75%) was significantly higher than that in PAAD tissues (Staining: Low, Intensity: Weak, Quantity: 75%-25%). VEGFA staining in normal pancreatic tissue (Staining: High, Intensity: Strong, Quantity:>75%) was also significantly higher than in PAAD tissue (Staining: Low, Intensity: Weak, Quantity: >75%). Interestingly, however, the immunohistochemical results of SOD1 were not completely consistent with the differential expression analysis. SOD1 was lowly expressed in exocrine cells of normal pancreatic tissues (Staining: Low, Intensity: Weak, Quantity:>75%); moderately expressed in endocrine cells (Staining: Medium. Intensity: Moderate, Quantity:>75%); but also generally low in PAAD tissues (Staining: Low, Intensity: Weak, Quantity: >75%). We speculate that the inconsistency is related to the overall low expression of SOD1.

### 3.2 Immune landscape of differentially expressed CRGs

**Figure 3:**
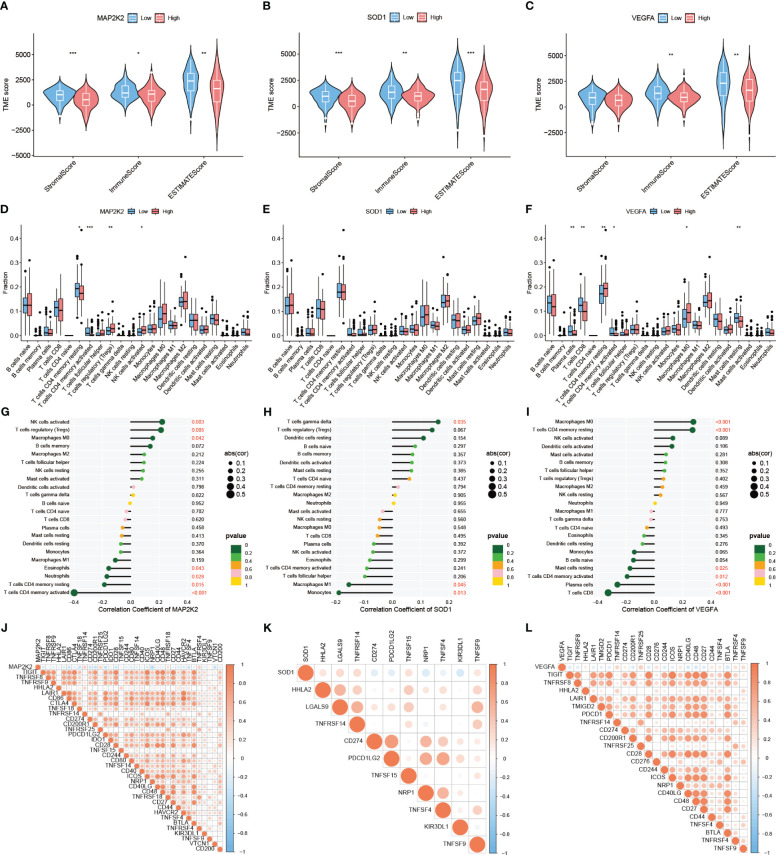
Immune landscape of differentially expressed CRGs. TME evaluation **(A–C)** and immune cell infiltration **(D–F)** of MAP2K2, SOD1, and VEGFA high and low expression groups ("***": P<0.001, "**": P<0.01, "*": P<0.05). Correlation of MAP2K2, SOD1, and VEGFA expression profiles with immune cells **(G–I)** and 47 immune checkpoints **(J–L)**.

Based on the screening of 3 genes that were differentially expressed at the PAAD transcriptome level, their immune microenvironment was further analyzed in the TCGA-PAAD cohort. The high and low expression groups of MAP2K2, SOD1, and VEGFA were first scored for TME (**Figure 3A-C**). The results showed that the Stromal Score, Immune Score, and ESTIMATE Score of the low expression group of these 3 genes were higher than the high expression group. This indicates that for MAP2K2, SOD1, and VEGFA, the stromal cell component and immune cell component were higher in the low expression group than in the high expression group, suggesting that immunosuppressive microenvironment was prevalent in the high expression group. Interestingly, the expression of MAP2K2 and VEGFA did not match the TME scoring. To further understand the specific composition of TME in the high- and low-expression groups, we analyzed the immune cell infiltration of the three CRGs (**Figure 3D-F**). The results showed that T cells CD4 memory resting and T cells CD4 memory activated were mainly distributed in the MAP2K2 low expression group; T cells regulatory (Tregs) and NK cells activated were mainly distributed in the MAP2K2 high expression group. Plasma cells, T cells CD8, T cells CD4 memory activated, and Mast cells resting were mainly distributed in the low VEGFA expression group; Macrophages M0 and T cells CD4 memory resting were mainly distributed in the high VEGFA expression group. And the difference of SOD1 was not significant. To judge the accuracy of immune cell infiltration analysis, the correlation between MAP2K2, SOD1, and VEGFA with immune cells was analyzed separately (**Figure 3G-I**), and the results were consistent with previous. Among them, Macrophages, as the immune cell component associated with all three CRGs, Macrophages M0 was positively correlated with the expression of MAP2K2 (Cor=0.1557, P=0.0420) and VEGFA (Cor=0.2755, P=0.0003), and Macrophages M1 was negatively correlated with the expression of SOD1(Cor=-0.1538, P=0.0446). The 47 immune check loci were weakly to moderately correlated with MAP2K2, SOD1, and VEGFA (**Figure 3J-L**).

### 3.3 Expression patterns of immune-related CRGs at single-cell level

**Figure 4:**
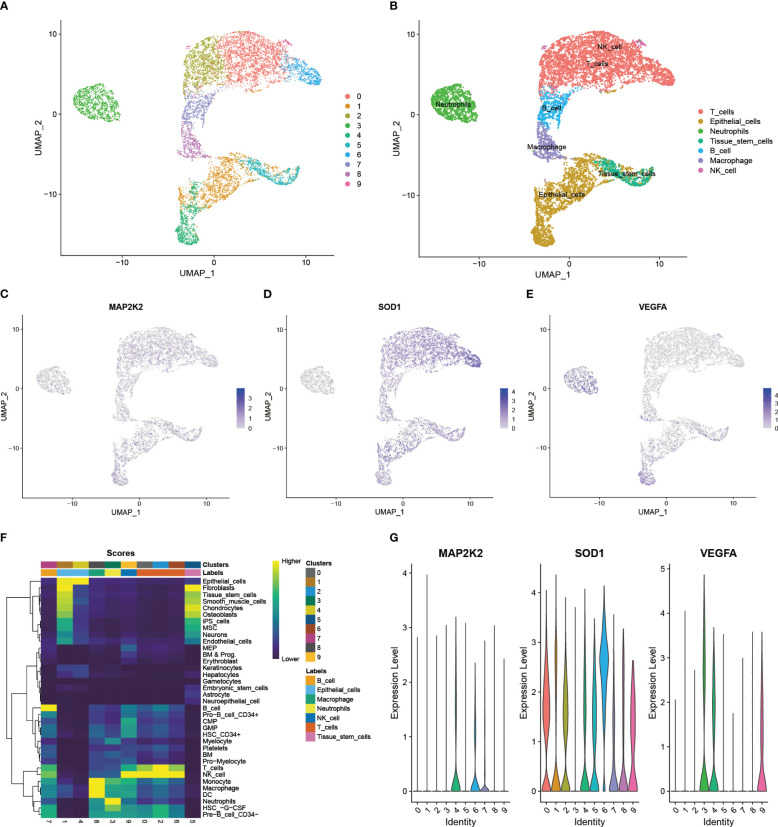
Expression patterns of immune-related CRGs at single-cell level. **(A)** 10 major cell clusters in the scRNA-PAAD cohort **(B)** 7 cell types identified based on cellular markers: Epithelial cells, Tissue stem cells, T cells, Neutrophils, B cells Macrophages, and NK cells **(C-E)** Expression patterns of the differentially expressed 3 CRGs in cancer tissues: MAP2K2, SOD1, and VEGFA **(F)** Accuracy of cell cluster annotation identification **(G)** Specific expression levels of the 3 CRGs in each cell cluster.

All 6 cancer tissue samples from 6 PAAD patients of GSE212966 participated in this study. After quality control (200< nFeatureRNA< 2500, per cent.mt< 5), a total of 9431 single cells and 28435 genes were included in the study for further analysis (**Supplementary Figure S1A, 1B**). Normalization was performed on the basis of the first 2000 highly variable genes, of which the first 10 genes are presented in the figure (**Supplementary Figure S1C**). The key genes and their importance in the first 2 principal components exhibited satisfactory between-group variability (**Supplementary Figure S1D**). As shown, these cells could be divided into 10 major cell clusters (**Figure 4A**). Among them, the first 5 marker genes of each cell cluster had significant intergroup differences (**Supplementary Figure S1E**), and these marker genes were the basis of the annotation. By annotation, these 10 cell clusters were clearly grouped into 7 categories (**Figure 4B**). In addition to Epithelial cells, which contain cancer cells, Tissue stem cells and 5 types of immune cells (T cells, Neutrophils, B cells, Macrophages, and NK cells) are also present. By comparing the expression patterns of CRGs in single cells, 19 of these genes were found to be significantly expressed in PAAD samples. Among them, COX17, FDX1, GLS, MT1E, MT1F, MT1G, MT1X, MT2A, and PDE3B were mainly clustered in NK cell clusters (**Supplementary Figure S2A**). VEGFA was mainly clustered in Neutrophils clusters (**Supplementary Figure S2B**). ATOX1 was mainly clustered in Macrophages (**Supplementary Figure S2C**). MAP2K2 was mainly clustered in Epithelial cells (**Supplementary Figure S2D**). SOD1, MT-CO2, UBE2D2, and UBE2D3 were commonly and significantly expressed in PAAD monocytes (**Supplementary Figure S2E**). Next, to further explore the differentially expressed 3 CRGs at the transcriptome level, we performed an in-depth analysis of their specific expression levels and distribution. Among them, MAP2K2 was mainly expressed in Epithelial cells, B cells, and T cells clusters (**Figure 4C**), SOD1 was mainly expressed in Endothelial cells and T cells clusters (**Figure 4D**), and VEGFA was mainly expressed in Endothelial cells, Neutrophils and T cell clusters (**Figure 4E**). The annotation accuracy was checked and the distribution was verified by quantifying the expression (**Figure 4F, G**), which was consistent with the counts results. In addition to this, the paraneoplastic normal 3-case samples demonstrated satisfactory quality under the same QC treatments described above (**Supplementary Figure S3**). The patterns of 3 differentially expressed immune-associated CRGs, MAP2K2, SOD1, and VEGFA, in 3 single-cell samples of normal tissues adjacent to cancer were further analyzed as controls (**Supplementary Figure S4A-S4B**). As seen in the figure, MAP2K2 was mainly expressed in NK cells and CMP clusters (**Supplementary Figure S4C**), SOD1 was commonly and significantly expressed in all clusters (**Supplementary Figure S4D**), and VEGFA was mainly expressed in monocytes and CMP clusters (**Supplementary Figure S4E**). These results were validated using the same method as above (**Supplementary Figure S4F-S4G**). The single-cell results of PAAD showed more pronounced infiltration of B cells, macrophages, NK cells, and T cells compared to normal tissues, which suggests that the inflammatory features of PAAD seem to change with disease progression. Furthermore, single-cell sequencing data of the 9-case PAAD samples and their adjacent 9-case normal tissue samples from GSE205049 were used as external validation and support for the above single-cell analysis results. The analysis results showed that the 9 PAAD samples could be annotated into 5 cell clusters (**Supplementary Figure S5A, 5B**), whereas the 9 normal para-cancerous samples could be annotated into 7 cell clusters (**Supplementary Figure S6A, 6B**). MAP2K2 was expressed predominantly in the NK cell, T cell, and monocyte clusters (**Supplementary Figure S5C**). SOD1 was commonly and significantly expressed across all the clusters (**Supplementary Figure S5D**). VEGFA was expressed mainly in the monocyte and NK cell clusters (**Supplementary Figure S5E**). Compared to normal tissues (**Supplementary Figure S6C-6E**), the single-cell results of PAAD showed more dense and more pronounced immune cells infiltration (T cells, B cells, NK cells and monocytes) under the same filtration conditions. This is consistent with the results of the GSE212966 analysis. Annotation accuracy heatmaps and expression distribution violin plots demonstrated satisfactory reliability of the conclusions (**Supplementary Figures S5F, 5G, 6F, 6G**). And the differential distribution of CRGs at the single cell level in PAAD and its adjacent normal tissues suggests that the occurrence of Cuproptosis is not only associated with the cancer cells themselves but also with the type of immune cells infiltrating the inflammatory TIME in PAAD. However, the exact mechanism needs to be further explored.

### 3.4 Establishment of Cuproptosis-related signature to assess individual prognosis

**Figure 5:**
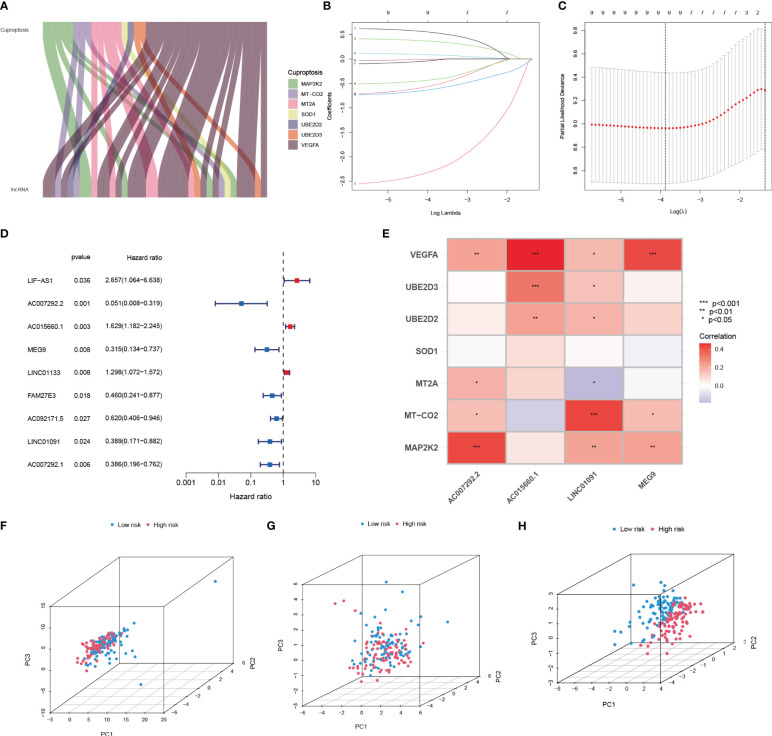
Establishment of Cuproptosis-related signature to assess individual prognosis. **(A)** Co-expression relationship of 7 CRGs with CRIs **(B, C)** Lasso-Cox regression analysis **(D)** Univariate COX regression analysis demonstrating 9 CRIs and their P value and Hazard Ratio **(E)** Risk lncRNAs of CIR-score and 7 CRGs correlation **(F-H)** PCA analysis according to CRGs, CRIs, and CIR-score.

A total of 37 CRIs were identified in PAAD based on the 7 CRGs (**Supplementary Table S6**) that had interactions with IRGs (|PearsonR| > 0.4 and p< 0.001). And most of the CRGs were positively correlated with CRIs (**Figure 5A**, **Supplementary Table S7**). 9 differentially expressed CRIs with prognostic value were obtained by Lasso-Cox regression (**Figure 5B, C**) and one-way regression analysis (**Figure 5D**), namely, LIF-AS1, AC007292.2, AC015660.1, MEG9, LINC01133, FAM27E3, AC092171.5, LINC01091, and AC007292.1. Based on these 9 CRIs and multi-factor regression analysis, 4 key CRIs were extracted to construct the signature, including AC007292.2, AC015660.1, LINC01091, and MEG9 (**Figure 5E**). We named this signature of 4-CRIs as CIR-score (**Table 1**). In addition, we respectively performed PCA downscaling analysis of the TCGA-PAAD cohort according to CRGs, CRIs, and risk lncRNAs of CIR-score (**Figure 5F–H**). The results showed that the descending results conditional on the CIR-score were significantly better than the other two groups, validating the reliability of the CIR-score.

**Table 1:** Hub lncRNAs and their correlation coefficients of CIR-score.

lncRNA	Coef
AC007292.2	-2.8982676561
AC015660.1	0.4969020144
LINC01091	-0.7786642720
MEG9	-0.8524089223

### 3.5 Validation of the independent predictive power of the CIR-score to assess individual prognosis

**Figure 6:**
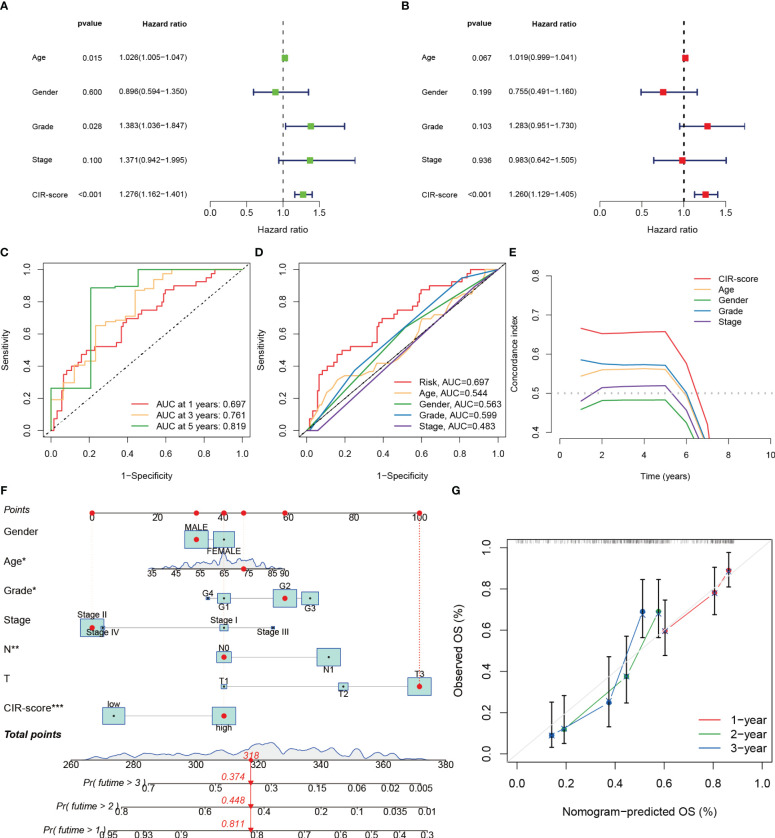
Validation of the independent predictive power of the CIR-score to assess individual prognosis. **(A, B)** Univariate and multifactorial Cox analysis considering the CIR-score and clinical characteristics **(C)** ROC curves for 1-, 3- and 5-year overall survival **(D)** ROC curves for the CIR-score and other clinical indicators **(E)** CIR-score and other clinical indicators c- index curves **(F)** A Nomogram that predicts 1-, 2-, and 3-year survival in patients with PAAD ("***": P<0.001, "**": P<0.01, "*": P<0.05) **(G)** Calibration curves for this Nomogram.

Whether the CIR-score is an indicator with independent prognostic value was explored by performing univariate and multifactorial Cox regression analyses in the training set of TCGA-PAAD (**Figure 6A, B**). The results showed that in the univariate Cox regression analysis, age (p=0.015, Hazard Ratio=1.026), Grade (p=0.028, Hazard Ratio=1.383), and CIR-score (p<0.001, Hazard Ratio=1.276) of PAAD patients were significantly associated with prognosis correlation. The ROC curve showed good results in evaluating the survival of PAAD patients at 1, 3, and 5 years (**Figure 6C**). Additionally, the AUC of CIR-score was the highest compared to the AUC of age, gender, tumor grade, and clinical stage at 0.697 (**Figure 6D**). C-index curves showed the same results as the former (**Figure 6E**). This evidence suggests that the CIR-score has a better ability to predict prognosis compared with other clinical characteristics. On this basis, we constructed a Nomogram based on the CIR-score and other clinically relevant indicators to assess the 1-, 2-, and 3-year survival of individual patients (**Figure 6F**). Its calibration curve also confirmed its good predictive power (**Figure 6G**).

### 3.6 Prognostic power and clinical relevance of CIR-score

**Figure 7:**
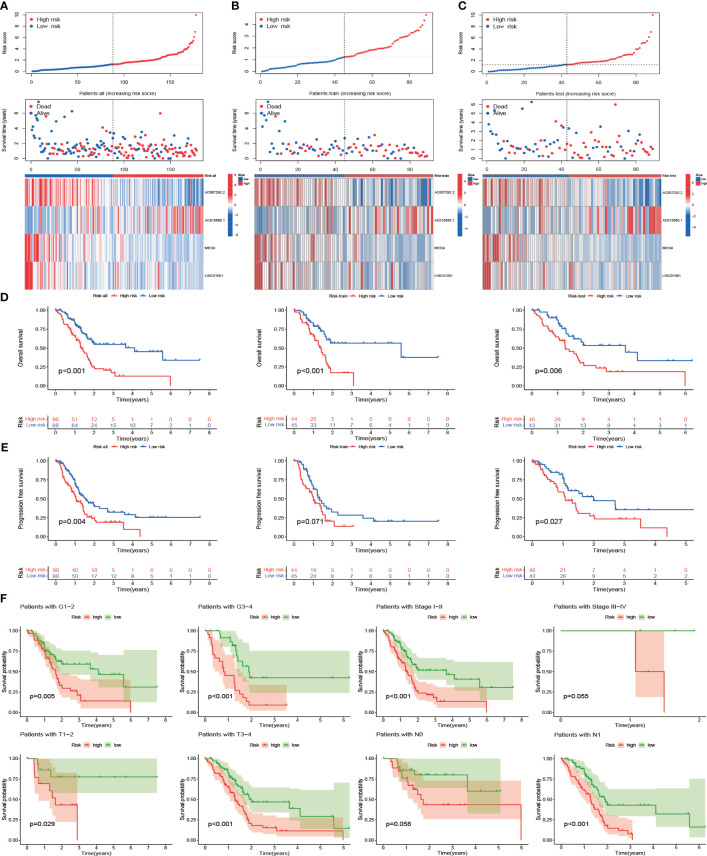
Prognostic power and clinical relevance of CIR-score. **(A-C)** Standardized distribution of scores, distribution of survival time and survival status, and differences in expression of risk lncRNAs in the TCGA-PAAD cohort, train set, and test set with high- and low-risk **(D)** Survival analysis of these 3 groups **(E)** Differences in progression-free survival of these 3 groups **(F)** Survival analysis of the high- and low-risk groups for different clinical indicators.

Based on the CIR-score, samples from the TCGA-PAAD cohort, train set, and test set were scored separately and divided into high- and low-risk groups based on the median (**Supplementary Table S8**). No between-group differences in clinical risk factors between the train and test sets demonstrated the reliability of the randomized groupings (**Supplementary Table S9**). The standardized distribution of CIR-score, scatter plots of survival time and survival status, and heat maps of expression of risk lncRNAs were plotted for the high- and low-risk groups, respectively (**Figure 7A-C**). The results showed that the survival time and survival rate were significantly higher in the low-risk group than in the high-risk group. The differences in the expression levels of the 4 risk lncRNAs of the CIR-score were also consistent with each other in the high- and low-risk groups. Furthermore, survival analysis and Kaplan-Meier curves were performed on samples from the TCGA-PAAD cohort, train set, and test set respectively (**Figure 7D**). As expected, PAAD cases with higher CIR-score obtained significantly worse OS in the TCGA-PAAD cohort (p< 0.001), train set (p< 0.001), and test set (p=0.006). This is consistent with Progression Free Survival (PFS) in the TCGA-PAAD cohort (p= 0.004), and test set (p= 0.027) (**Figure 7E**). However, the variability of PFS in the train set was not significant (p= 0.071). We speculate that this is related to the size of the sample size. Then, the TCGA-PAAD cohort was subjected to CIR-score scoring, and the sample was divided into 2 groups of high and low risk according to the median. The TCGA-PAAD cohort was subgrouped according to different prognosis-related clinical traits, and survival analysis was performed for each group of samples (**Figure 7F**). Results showed that Survival probability was significantly higher in the low-risk group than in the high-risk group in the Grade 1-2 cohort (p= 0.005) and the Grade 3-4 cohort (p< 0.001). In the Stage I-II cohort (p< 0.001), higher risk scores were consistent with survival. In addition, in cohorts differentiated according to TNM staging, risk scores were consistent with survival in the T1-2 cohort (p= 0.029), T3-4 cohort (p< 0.001), and N1 cohort (p< 0.001). Nevertheless, in the Stage III-IV cohort (p= 0.055) and N0 cohort (p= 0.058), the differences were not significant due to insufficient sample size or too many missing values.

### 3.7 Differences in the TIME landscape between high and low-risk groups

**Figure 8:**
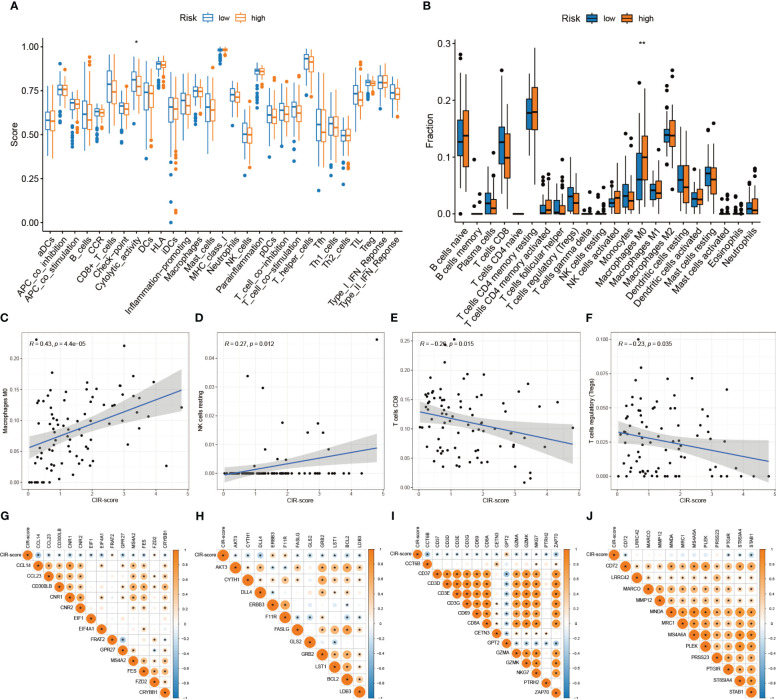
Differences in the TIME landscape between high and low-risk groups. **(A)** Differences between immune-related activities in high and low-risk groups ("*": P<0.05) **(B)** Immune cell infiltration in tumor microenvironment in high and low-risk groups ("**": P<0.01) **(C–F)** Correlation analysis between immune cells (Macrophages M0, NK cells resting, T cells CD8, T cells regulatory) and CIR-score **(G–J)** Correlation analysis between immune checkpoints (Macrophage, NK cells, Activated CD8 T cells, Regulatory T cells) and CIR-score.

Correlation analysis of immune activity was performed for the high- and low-risk groups according to the CIR-score (**Figure 8A**). The results showed that the immune function scores of the low-risk group were generally higher than those of the high-risk group, suggesting a significant state of immunosuppression in the high-risk group. Next, the fraction of all 22 immune cells in the high- and low-risk groups was explored (**Figure 8B**). The most significant intergroup variability was found for Macrophages M0, NK cells resting, T cells CD8, and T cells regulatory (Tregs). Among them, Macrophages M0 and NK cells resting were more significantly infiltrated in the high-risk group. T cells CD8 and T cells regulatory were more significantly infiltrated in the low-risk group. That is, Macrophages M0 (R = 0.43, p = 4.4e-05) and NK cells resting (R = 0.27, p = 0.012) were positively correlated with CIR-score, and T cells CD8 (R = -0.26, p = 0.015) and T cells regulatory (R = - 0.23, p = 0.035) showed a negative correlation with CIR-score (**Figure 8C-F**). Besides, by summarizing the immune checkpoint genes of different immune cells (26), we further explored the correlation of CIR-score with these four immune microenvironment cells (**Figure 8G-J**). The results showed that the CIR-score was moderately correlated with the immune checkpoints of Macrophage, Natural killer cell, Activated CD8 T cell, and Regulatory T cell.

### 3.8 Enrichment analysis, tumor burden, and immune checkpoint sensitivity prediction

**Figure 9:**
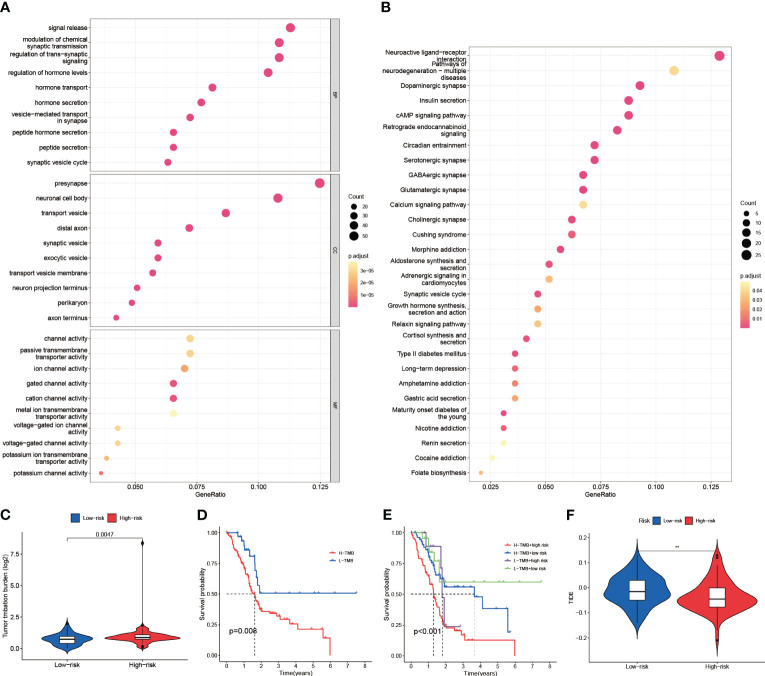
Enrichment analysis, tumor burden, and immune checkpoint sensitivity prediction. **(A)** Results of GO enrichment analysis (Biological Process, Cellular Components, and Molecular Function) **(B)** Results of KEGG enrichment analysis (Pathways) **(C)** TMB differences between high and low-risk groups (D) High and low TMB groups for survival analysis **(E)** Survival analysis of high TMB-high risk group, high TMB-low risk group, low TMB-high risk group, and low TMB-low risk group **(F)** TIDE assessment of high and low-risk groups ("**": P<0.01).

To further analyze the intergroup differences between high and low-risk groups, DEGs of high and low-risk groups were screened. Then, GO and KEGG enrichment analyses were performed on these DEGs. In GO analysis (**Supplementary Table S10**), DEGs were mainly enriched in signaling as well as hormone-related biological processes (BP), cytological components (CC) such as presynapse, and molecular biological functions (MF) related to multiple channel activities (**Figure 9A**). In KEGG analysis (**Supplementary Table S11**), Neuroactive ligand-receptor interaction, Pathways of neurodegeneration-multiple diseases, and Dopaminergic synapse related pathways were represented in a larger proportion (**Figure 9B**). Therefore, we hypothesized that DEGs mainly function in cell signaling and hormonal regulation. Next, we assessed Tumor Mutation Burden (TMB) in the high- and low-risk groups (**Figure 9C, D**). The high-risk group had a higher TMB compared to the low-risk group (p=0.0047). And the high TMB group had a significantly worse prognosis than the low-risk group (p=0.008). By further analysis of the high-low risk group versus the high-low TMB group (**Figure 9E**), it was obtained that the CIR-score had a stronger potential to predict survival than TMB (p<0.001). Additionally, to explore the potential clinical efficacy of immunotherapy in different subgroups, the Tumor Immune Dysfunction and Exclusion score (TIDE) was applied (**Figure 9F**). The TIDE prediction score was significantly higher in the low-risk group than in the high-risk group, indicating that patients in the low-risk group were more likely to experience immune escape and patients were less likely to benefit from Immune Checkpoint Inhibitor (ICI) therapy (p<0.01). However, given the uncertainty of TIDE for tumor scores other than the melanoma dataset and the non-small cell lung cancer dataset, we still need to make further predictions about the likelihood of drug treatment in the high- and low-risk groups.

### 3.9 Drug sensitivity intergroup differences

**Figure 10:**
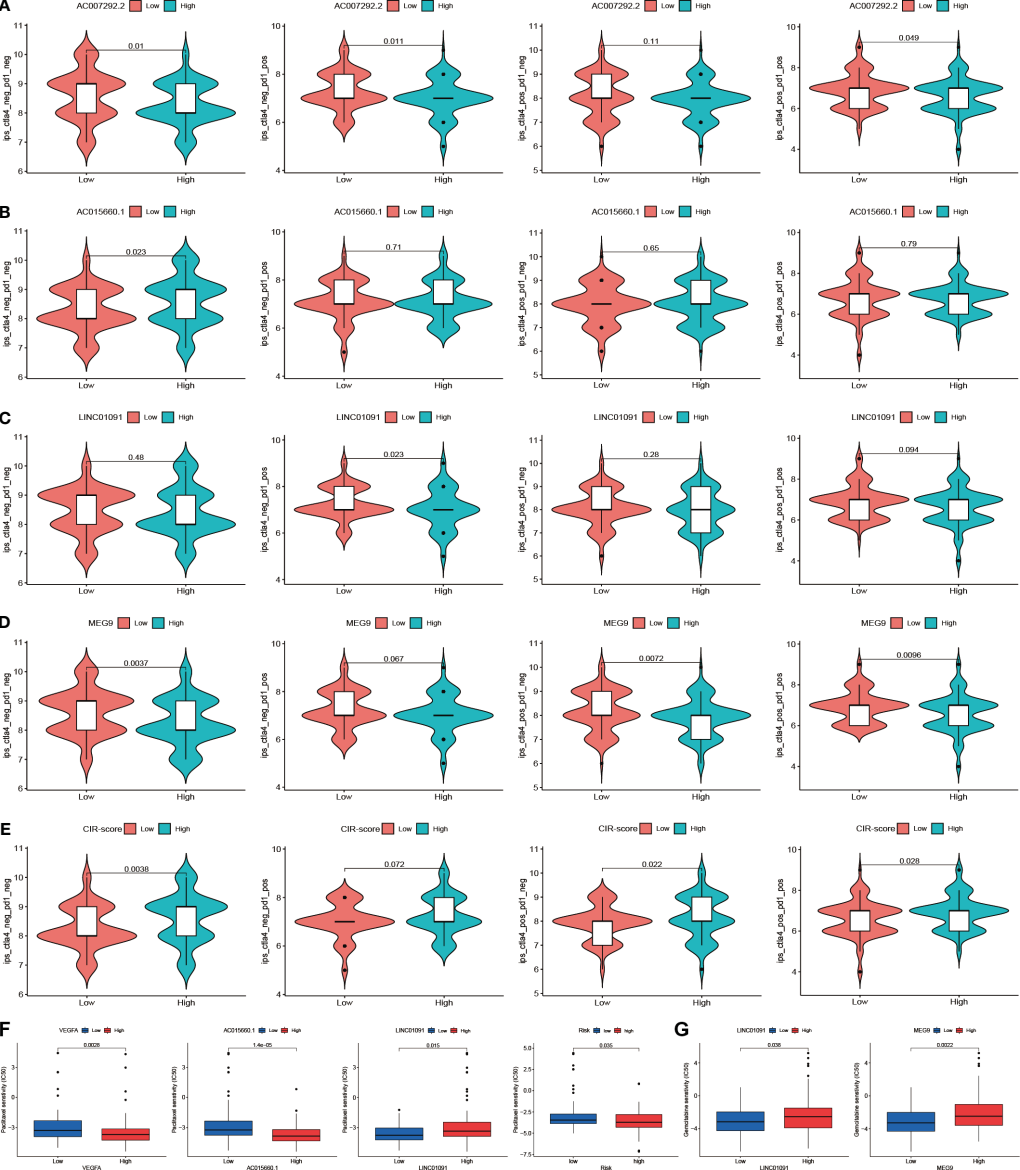
Drug sensitivity intergroup differences. **(A-D)** Immunogenicity differences of the 4 hub lncRNAs: AC007292.2, AC015660.1, LINC01091, and MEG9 with different expression **(E)** Immunogenicity differences between the high and low-risk groups **(F)** IC50 differences of Paclitaxel in VEGFA, AC015660.1, LINC01091, and CIR-score with different levels **(G)** IC50 differences of Gemcitabine in LINC01091 and MEG9 with different levels.

To deeply explore the different focus of drug therapy in patients in high and low-risk groups. The Immune cells Proportion Score (IPS) differences between the high and low expression groups of CRGs and CRIs and the high and low-risk groups of CIR-score were evaluated based on the expression levels of hub CRGs and lncRNAs, and the differences in immunotherapy sensitivity between the different groups were compared. Higher IPS was associated with higher immunogenicity. The results indicated that although the differences in PD-1 were not significant in any of the 3 CRGs high and low expression groups. Nevertheless, for hub lncRNAs, the AC007292.2 low expression group (**Figure 10A**), AC015660.1 high expression group (**Figure 10B**), LINC01091 low expression group (**Figure 10C**), MEG9 low expression group (**Figure 10D**), and CIR-score high-risk group (**Figure 10E**) possessed significantly higher immunogenicity. Moreover, the difference in PD-1 monoclonal antibody sensitivity between the CIR-score high- and low-risk groups was far more significant than that of single hub lncRNA, which demonstrated that CIR-score is more significant for guiding immunotherapy dosing strategies than single hub lncRNA and single CRG. In addition, considering that Nanoalbumin Paclitaxel (Abraxane^®^) in combination with Gemcitabine is the preferred chemotherapy for non-surgical PAAD patients (27), we further probed into the intergroup differences in the IC50 of these two agents. A lower IC50 was associated with higher drug sensitivity. Paclitaxel was found to be more sensitive in the VEGFA high expression group, AC015660.1 high expression group, LINC01091 low expression group, and CIR-score high-risk group (**Figure 10F**). Gemcitabine was more sensitive in the LINC01091 low expression group, and MEG9 low expression group (**Figure 10G**). The above results suggest that patients with lower CIR-score are more likely to be resistant to immunotherapy and chemotherapy.

### 3.10 Validation of hub CRGs and lncRNAs in immune combined targeted and chemotherapy mouse PAAD model

**Figure 11:**
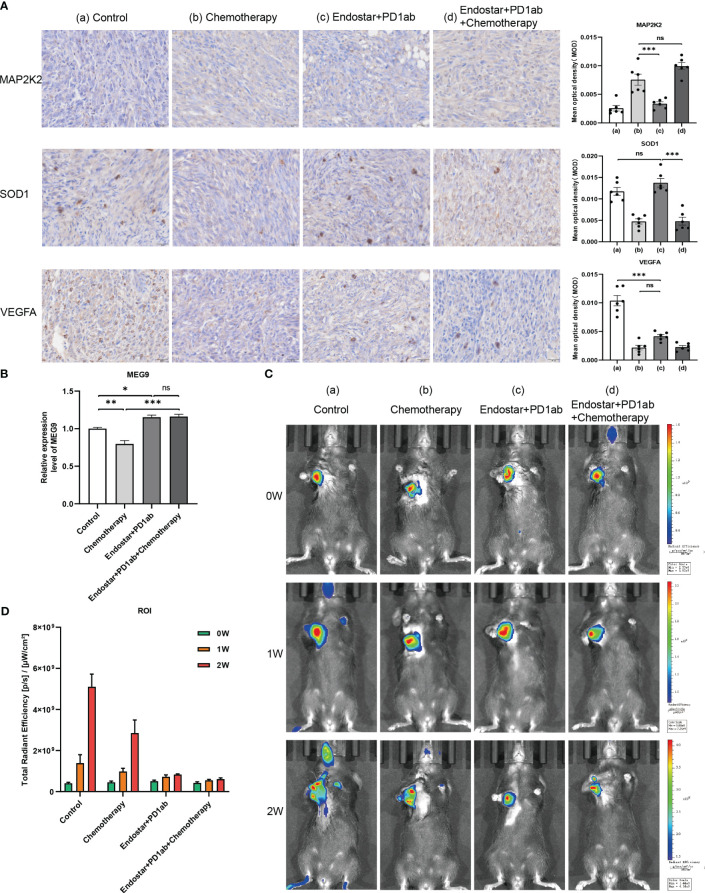
Validation of hub CRGs and lncRNAs in immune combined targeted and chemotherapy mouse PAAD model. **(A)** Immunohistochemical results of 3 differentially expressed immune-related CRGs in mouse PAAD model (a: Model Control Group; b: Chemotherapy Group; c: Recombinant human endostatin + PD-1 Monoclonal Antibody Group; d: Recombinant human endostatin + PD-1 Monoclonal Antibody + Chemotherapy Group) **(B)** Hub lncRNA MEG9 relative expression differences in the 4 treatment cohorts ("***": P<0.001, "**": P<0.01, "*": P<0.05) **(C)** *In vivo* imaging of PANC-02 transplanted tumors in different treatment cohorts of PAAD mice at 0, 1, and 2 weeks **(D)** Differences in total radiation efficiency in different treatment cohorts at 0, 1, and 2 weeks.

The clinical benefit of monotherapy is limited to a small population. Currently, the efficacy of immunotherapy (PD-1 monoclonal antibody) alone plus chemotherapy (28) and immunotherapy combined with other molecules is suboptimal (29). In contrast, chemotherapy combined with recombinant human vascular endothelial inhibitor (Endostar ^®^) has achieved some achievements in clinical treatment (30). Therefore, to explore new strategies for combination therapy, 4 PAAD mouse treatment cohorts of model control group, chemotherapy group (Nanoalbumin Paclitaxel + Gemcitabine), recombinant human vascular endothelial inhibitor + PD-1 monoclonal antibody group, and recombinant human vascular endothelial inhibitor + PD-1 monoclonal antibody + chemotherapy group were included in this study. Although we assessed the efficacy of immunotherapy in patients by screening immune-related CRGs as raw material for the construction of CIR-score and characterization, it is necessary to directly compare the response rates of hub CRGs and lncRNAs under different treatment regimens in a mouse PAAD model. Considering that MAP2K2 is positively correlated with AC007292.2 (Coef. = -2.898), LINC01091 (Coef. = -0.779), and MEG9 (Coef. = -0.852) (**Figure 5E**), indicating that the lower MAP2K2 expression had a higher CIR-score. Similarly, SOD1 was mildly negatively correlated with AC007292.2, LINC01091, and MEG9, and mildly positively correlated with AC015660.1 (Coef. = 0.497). VEGFA is positively correlated with AC007292.2, AC015660.1, LINC01091, and MEG9, but the correlation coefficient with AC015660.1 is the highest. That is, the higher the expression of SOD1 and VEGFA the higher the CIR-score. Therefore, the immunohistochemical results showed that the recombinant human vascular endothelial inhibitor + PD-1 monoclonal antibody + chemotherapy group had the lowest CIR-score, the model control group had the highest CIR-score, and the recombinant human vascular endothelial inhibitor + PD-1 monoclonal antibody group was slightly higher than the chemotherapy alone group (**Figure 11A**). In contrast, with almost all current drugs, cancer tissue becomes resistant over time as patients use them (31). Compared to those already on the drug, cancer patients who did not experience treatment were more sensitive to the same drug. And the high-scoring group was more sensitive to immunotherapy (**Figure 10E**). Thus, the differences in MAP2K2, SOD1, and VEGFA expression in different treatment cohorts were consistent with intergroup differences in immunotherapy sensitivity. Furthermore, MAP2K2 expression was significantly higher in the chemotherapy group than in the non-chemotherapy group, and SOD1 and VEGFA expression was significantly lower in the chemotherapy group than in the non-chemotherapy group. That is, the scores in the chemotherapy group were significantly lower than those in the non-chemotherapy group. This is also consistent with the result that the higher-scoring group was more sensitive to chemotherapy (**Figure 10F, G**). On the other hand, qRT-PCR results showed that the expression of MEG9 was significantly higher in the immunotherapy cohort in the presence of PD-1 monoclonal antibody treatment than in the non-immunotherapy cohort (**Figure 11B**), which is also consistent with the analysis of sensitivity differences between immunotherapy groups. Unfortunately, we did not retrieve the sequences of AC007292.2, AC015660.1, and LINC01091 in mice, and therefore did not validate them by qRT-PCR in mouse models. These 3 lncRNAs will be planned to be validated on human PAAD tissues in the future. The above results further demonstrate that CIR-score is qualified to predict the efficacy of immunotherapy in PAAD patients and provide guidance for individualized and precise application of immunotherapy. Interestingly, *in vivo* imaging showed that the Total Radiant Efficiency of recombinant human vascular endothelial inhibitor + PD-1 monoclonal antibody + chemotherapy group was significantly lower than the other 3 groups after 1 and 2 weeks of administration (**Figure 11C, D**). It indicates that immunotherapy combined with targeted and chemotherapy significantly improved the proliferation and progression of PAAD, which provides a new possibility for a multidrug combination therapy strategy for PAAD with immunotherapy as the main treatment.

## 4 Discussion

Cuproptosis is a novel cell death mechanism different from the known ones. Copper ions can mediate cell death through a mitochondria-dependent increase in energy metabolism and cytotoxicity induced by reactive oxygen species (ROS) accumulation (10). Copper deficiency can lead to early embryonic death or congenital malformations. This is due to copper’s role as a cofactor for mitochondrial cytochrome c oxidase, which is required to meet the energy requirements of rapidly dividing cells (24). Similarly, the copper requirement of cancer cells is higher compared to non-dividing cells. First, in studies of PAAD (11), gastric cancer (32), hepatocellular carcinoma (33), gallbladder cancer (34), lung cancer (35), thyroid cancer (36), and prostate cancer (37), serum copper ion levels were found to be significantly higher in tumor patients compared to normal patients. Higher serum copper ion concentrations in lung cancer (35) and breast cancer (38) were also associated with poorer clinical prognosis. Second, copper also reflects TIME status at the immune cell level. Copper ion concentration is inversely correlated with the degree of infiltration of T cells and macrophages in TIME. In mesothelioma, reducing bioavailable copper slows tumor growth, normalizes blood vessels, and promotes T-cell infiltration (39). In hepatocellular carcinoma, copper with disulfiram inhibits T-cell infiltration by inhibiting PARP1 activity and enhancing GSK3β Ser9 site phosphorylation, which in turn upregulates PD-L1 expression (40). In breast cancer, the copper-depleting compound tetrathiomolybdate (TM) resulted in reduced collagen deposition, decreased levels of myeloid-derived suppressor cells, and increased infiltration of CD4+ T cells (41). In addition, downregulation of the copper-containing metabolism MURR1 structural domain 1 (COMMD1) in cancer cells enhances the inflammatory response and creates favorable conditions for macrophage recruitment (42). Therefore, the present study screened for immune-related CRGs by exploring the interaction between IRGs and CRGs. The results revealed that among the 14 CRGs that interacted significantly with 35 IRGs, VEGFA, SOD1, and MAP2K2 had the most significant differential expression between PAAD and adjacent normal tissues. Among them, VEGFA encodes vascular endothelial growth factor A, which induces angiogenesis, vascular endothelial cell growth, and increased vascular permeability. Although VEGFA is upregulated in many tumors and is a target for many cancer therapies. However, approximately 90% of PAAD are ductal adenocarcinomas with KRAS mutations as the primary driver, which are typically characterized by fibrous tissue hyperplasia and reduced vascularity, which in turn leads to immunosuppression and resistance to chemotherapy and immunotherapy (43). Thus, the low expression of VEGFA in PAAD tissues is consistent with the low vascular density characteristic of cancer tissues. Copper-zinc superoxide dismutase encoded by SOD1 is one of the predominant intracellular antioxidant enzymes and an important target for cancer drug design. Overexpression of SOD1 in PAAD protects cancer cells from oxidative stress (44). Hence, SOD1 is an important means of regulating superoxide anion radical (O2–) and hydrogen peroxide (H2O2) levels in cancer cells. However, most of the reported SOD1 inhibitors such as ATN-224 (45), DDC (46), and LD100 (47) have shortcomings like inefficient inhibition or lack of selectivity for other copper-containing proteins. MAP2K2, one of the core drivers of cancer, encodes mitogen-activated protein kinase kinase 2 (MEK2) which plays a key role in mitogenic growth factor signaling, and knockdown of MEK2 inhibits the invasive ability of PAAD cancer cells (48). Interestingly, however, the expression level of MAP2K2 in PAAD tissues was not exactly consistent with previous studies. We speculate that this correlates with the cell subpopulation category in which MAP2K2 is highly expressed. To further explain the high expression of MAP2K2 in PAAD paracancerous tissues and to investigate the differences in the spatial distribution of CRGs expression levels, the present study further explored the expression patterns of all 14 CRGs with co-expression relationships with IRGs at the single-cell level.

The transformation of normal pancreatic follicular cells to PAAD is often accompanied by the appearance of chronic inflammation, which induces and chemotacticizes a large number of immune cells distributed in the dense pancreatic interstitial tissue. These immune cells undergo altered functions in TIME, which eventually produce amplified immunosuppressive signals leading to immune escape of tumor cells and promoting tumorigenesis (49). Considering the complexity and highly suppressive nature of PAAD TIME, single-cell sequencing, as an emerging technology, provides a new perspective for the study of TIME. On the one hand, single-cell sequencing technology can better reveal the heterogeneity of tumor cells at the cellular and molecular levels, capture different tumor states, and provide new perspectives for understanding tumor drug resistance (50). On the other hand, cell clusters and cell states with similar gene expression profiles can be more thoroughly analyzed from a multi-omics perspective (genomics, transcriptomics, epigenomics, and proteomics) (51). To further visualize the differences in expression of CRGs at the cellular level between PAAD and its adjacent normal tissues, and to further explore the association of CRGs with different immune cell subpopulations in TIME, all screened CRGs were analyzed in depth in this study in single-cell sequencing samples. Among them, SOD1, MT-CO2, UBE2D2, and UBE2D3 were commonly and significantly expressed in each single cell subpopulation of PAAD, and the cluster with the highest number of aggregated genes was the NK cells cluster (**Supplementary Figure S2**). Overall, for the 3 immune-related CRGs that were significantly differentially expressed in the transcriptomic data, the single-cell data were consistent with the results of transcriptomic data analysis. Among them, SOD1 was significantly more expressed in each cell cluster in PAAD tissues than normal. Previous studies have shown that SOD1 overexpression not only protects cancer cells from oxidative stress but also suppresses pro-inflammatory immune responses in colitis by preventing oxidative stress (52). We speculate that similarly, SOD1 overexpression in TIME could inhibit the immune response by suppressing immune cells and their secreted cytokines and enzymes. Second, VEGFA expression in neutrophils, NK cells, and epithelial cells was significantly higher in PAAD tissues than normal. However, in CMP cell clusters the cancer VEGFA expression was significantly lower than normal. Moreover, MAP2K2, as an oncogene, plays a key role in mitogenic growth factor signaling. But single-cell sequencing analysis showed that MAP2K2 was significantly more expressed in NK and CMP cell subsets in normal pancreatic tissues adjacent to cancer than in PAAD tissues. We speculate that the main reason for the difference in MAP2K2 transcriptome data does not lie in the cancer cells in the tissues, but in the NK cells in the TIME.

lncRNAs are promising circulating biomarkers in cancer diagnosis and prognosis, and exceptional candidates for further therapeutic exploration. However, considering that individual genes or lncRNAs are not sufficient to achieve the desired prediction, a new form of indicator is needed to provide new strategies for individualized therapy. lncRNA signature is a risk indicator that can systematically predict cancer survival prognosis, immune landscape, and drug sensitivity by combining multiple lncRNA molecules as variables assigned with their respective coefficients. It can circumvent the limitations of individual lncRNAs in predicting prognosis, but also avoid the defects of interactions among lncRNAs. Currently, studies on the prognostic signature of Cuproptosis-associated lncRNAs have been conducted in tumors such as bladder cancer (53), head and neck squamous cell carcinoma (54), and gastric cancer (55). However, most studies have used co-expression analysis for screening of lncRNAs, but few studies have systematically explored the genes from which lncRNAs originate, as well as their distribution and expression differences at the single-cell level. Furthermore, the role of Cuproptosis-associated lncRNAs, in the regulation of PAAD and its immunotherapy is unclear. Therefore, this study screened for 7-CRGs based on single-cell sequencing, which were both co-expressed with IRGs and significantly expressed in single cells. The immune-related 4-CRGs with the most prognostic value were identified by co-expression and regression analysis: MEG9, LINC01091, AC015660.1, and AC007292.2. Among them, MEG9 could protect endothelial cells from DNA damage-induced cell death and predict cancer prognosis through the PI3K-AKT signaling pathway (56). In addition, MEG9 was significantly associated with hepatitis B virus (HBV) infection status in hepatocellular carcinoma (57), and epidermal growth factor receptor (EGFR) status in adenocarcinoma patients with non-small-cell lung cancer (NSCLC) (58). In the PAAD field, MEG9 is involved in the construction of m6A-related prognostic signature as hub lncRNA (59). LINC01091 can coordinate the microRNA-128-3p/ELF4/CDX2 axis and thus promote gastric cancer growth and metastasis by way of exosomes on one hand (60) and can act as hub lncRNA in DNA methylation-related prognostic signatures of prostate cancer (61). AC015660.1 is involved in the angiogenesis of PAAD (62) and inflammatory correlation of gastric cancer (63) as hub lncRNA of prognostic signatures. In contrast, AC007292.2 has no previous relevant studies. To verify the prognostic value, this study further discussed the survival, clinical relevance, bioenrichment, and gene mutations in the high- and low-risk groups according to the CIR-score median. The results showed that the CIR-score could more clearly classify PAAD patients into high- and low-risk clusters compared to CRGs or CRIs alone. And CIR-score also showed a significant positive correlation with risk factors such as pathological grade, clinical grade, and TMB.

Furthermore, differences in the immune landscape between the high and low-expression groups of CRGs and the high and low-risk groups of the CIR-score raised additional concerns. Immune cells, tumor-associated fibroblasts (CAFs), vascular system, and extracellular matrix (ECM) are the 4 major components that constitute the highly immunosuppressive microenvironment of PAAD (64). 2 of the major types of immune cells are most associated with immunosuppressive TIME in PAAD: tumor-associated macrophages (TAMs) and tumor-infiltrating T cells. Previous studies have confirmed that TAMs not only promote tumor initiation but also serve as central drivers of immunosuppressive TIME by expressing cell surface receptors, secreting cytokines, chemokines, and enzymes that regulate the recruitment and function of multiple immune cell subtypes (65). In this study, we found that macrophage M0 was positively correlated with the expression of MAP2K2 and VEGFA; macrophage M1 and monocytes were negatively correlated with the expression of SOD1. The expression of CRGs in macrophage clusters of PAAD single-cell samples was significantly higher than in normal tissues. M0 is the state in which macrophages are not activated. In contrast, activated macrophages have the typical activation phenotype M1 and the selective activation phenotype M2. M1 subtypes not only promote antitumor immune responses but also increase radiotherapy sensitivity (66, 67). Higher numbers of M2 subtypes are associated with larger PAAD tumor volumes, early liver and local recurrence, accelerated lymphatic metastasis, and shorter survival (68). However, activation of TAMs inhibits TLR-mediated M1 polarization (69). To address this feature Guiducci C et al. investigated a TLR9 ligand that induced a shift from the M2 phenotype to M1 in macrophages and resulted in tumor shrinkage (70). Thus, targeting macrophages provides a direction for improving TIME in PAAD. On the other hand, this study found significantly lower infiltration of regulatory T cells (Tregs) in the high-risk group than in the low-risk group. Zhang Y et al. suggested that depletion of Tregs accelerates pancreatic carcinogenesis, leads to differentiation of inflammatory fibroblast subpopulations, and consequently leads to compensatory immunosuppression in advanced disease (71). The more pronounced Tregs depletion and worse OS in the high-risk group are consistent with previous studies. This cross-talk between Tregs and fibroblasts in PAAD reveals a potential new therapeutic approach to alleviate immunosuppression in pancreatic cancer.

Immunotherapy is considered to be the fourth major oncology treatment after surgery, chemotherapy and radiotherapy. Although good efficacy has been achieved in some advanced tumors, the overall effect of immunotherapy in PAAD is poor. Compared with the poorer response to PD-1 inhibition alone, combination therapy with targeted chemotherapy and other agents has shown a more durable response, providing new ideas for the clinical treatment of PAAD (72). Recombinant human vascular endothelial inhibitor alleviates immunosuppression by normalizing tumor vasculature (73) and specifically replicates in PAAD cancer cells and kills them (74). PD-1 monoclonal antibody in combination with recombinant human vascular endothelial inhibitor and chemotherapy has shown good efficacy and safety in patients with advanced non-small cell lung cancer (NSCLC) (75). Combining recombinant human vascular endothelial inhibitor with first-line standard chemotherapy also improved the quality of life in patients with small cell lung cancer (30). However, no such combination therapy has been reported in the field of PAAD. Considering that Nanoalbumin Paclitaxel plus Gemcitabine is the chemotherapy of choice for PAAD patients who are non-surgical candidates but in good physical condition (27). And the CIR-score high and low-scoring groups and their associated high and low-expression groups of CRGs and CRIs exhibited significant drug sensitivity differences. To explore a more optimal combination treatment strategy for PAAD and to validate the reliability of the CIR-score, this study divided the mouse model into 4 treatment cohorts: model control group, chemotherapy group (Nanoalbumin Paclitaxel + Gemcitabine), recombinant human vascular endothelial inhibitor + PD-1 monoclonal antibody group, and recombinant human vascular endothelial inhibitor + PD-1 monoclonal antibody + chemotherapy group. MAP2K2, SOD1, and VEGFA, as the most prognostically valuable CRGs screened and co-expressed with CIR-score, were differentially expressed in the chemotherapy and non-chemotherapy groups in agreement with the pharmacoresensitivity prediction results (**Figure 10**). The qRT-PCR results of MEG9 in the hub lncRNAs of CIR-score were also consistent with the intergroup differences in immunotherapy sensitivity. These results further demonstrate that the CIR-score is qualified to predict immunotherapy efficacy in PAAD patients and provide guidance for individualized and precise application of immunotherapeutic agents. In addition, the recombinant human vascular endothelial inhibitor + PD-1 monoclonal antibody + chemotherapy group in the *in vivo* imaging results demonstrated a significant advantage in delaying tumor progression. Therefore, not only the CIR-score can predict the effect of immunotherapy in patients and reflect the difference of drug sensitivity in patients; but also immunotherapy combined with chemotherapy and targeted therapy can be a direction of clinical drug treatment. The combination of these two aspects suggests a focused multi-modality combination therapy for specific patients’ signature scores for the purpose of achieving optimal therapeutic outcomes, providing a new strategy for individualized treatment of PAAD.

There are also some limitations in this study. Firstly, unfortunately, we were unable to quantify all 4 hub lncRNAs by qRT-PCR in a mouse model because we did not retrieve the sequences of AC007292.2, AC015660.1, and LINC01091 in mice. These 3 IncRNAs will be planned to be further validated on human PAAD tissues in the future. Secondly, because of relying mainly on public databases and animal experiments, this study still needs to further investigate and validate of the value of prognostic CIR-score in clinical PAAD cases, the value of reflecting the immune landscape, and the value of guiding individual clinical medication use. For the future study and validation of CIR-score in human PAAD tissues, it is possible to assess the expression of CIR-score hub lncRNAs in biopsied tumor tissues of PAAD patients, and then bring the expression results into the CIR-score formula constructed in this study to assess the risk level of patients. The risk level of each patient can also be used as a reference index for the assessment of the patient’s immunotherapy sensitivity. In addition, the Nomogram constructed in this study can be used to individualize the survival rate of each patient based on the routine clinical risk factors collected from the patients and the CIR-score calculated in the clinic.

## 5 Conclusion

In conclusion, the construction and validation of a Cuproptosis-associated PAAD lncRNAs signature based on single-cell sequencing carried out in this study not only reveals the organizational principles that shape the TIME complexity of PAAD but also provides certain ideas for immunotherapy-based combination treatment strategies.

## Data availability statement

The datasets presented in this study can be found in online repositories. The names of the repository/repositories and accession number(s) can be found in the article/**Supplementary Material**.

## Ethics statement

The animal study was approved by Animal Ethics Committee of Jiangsu University. The study was conducted in accordance with the local legislation and institutional requirements.

## Author contributions

YF provided direction and guidance throughout the preparation of this manuscript. YS wrote and edited the manuscript. ZG, CM, and LY collected and prepared the related papers. RH and ZG reviewed and made significant revisions to the manuscript. All authors contributed to the article and approved the submitted version.
