# Supplementary figures and images for: Development and validation of cuproptosis-related lncRNAs associated with pancreatic cancer immune microenvironment based on single-cell
Source: Front Immunol. 2023 Sep 25;14:1220760. doi: 10.3389/fimmu.2023.1220760 (PMC10563513; doi:10.3389/fimmu.2023.1220760)

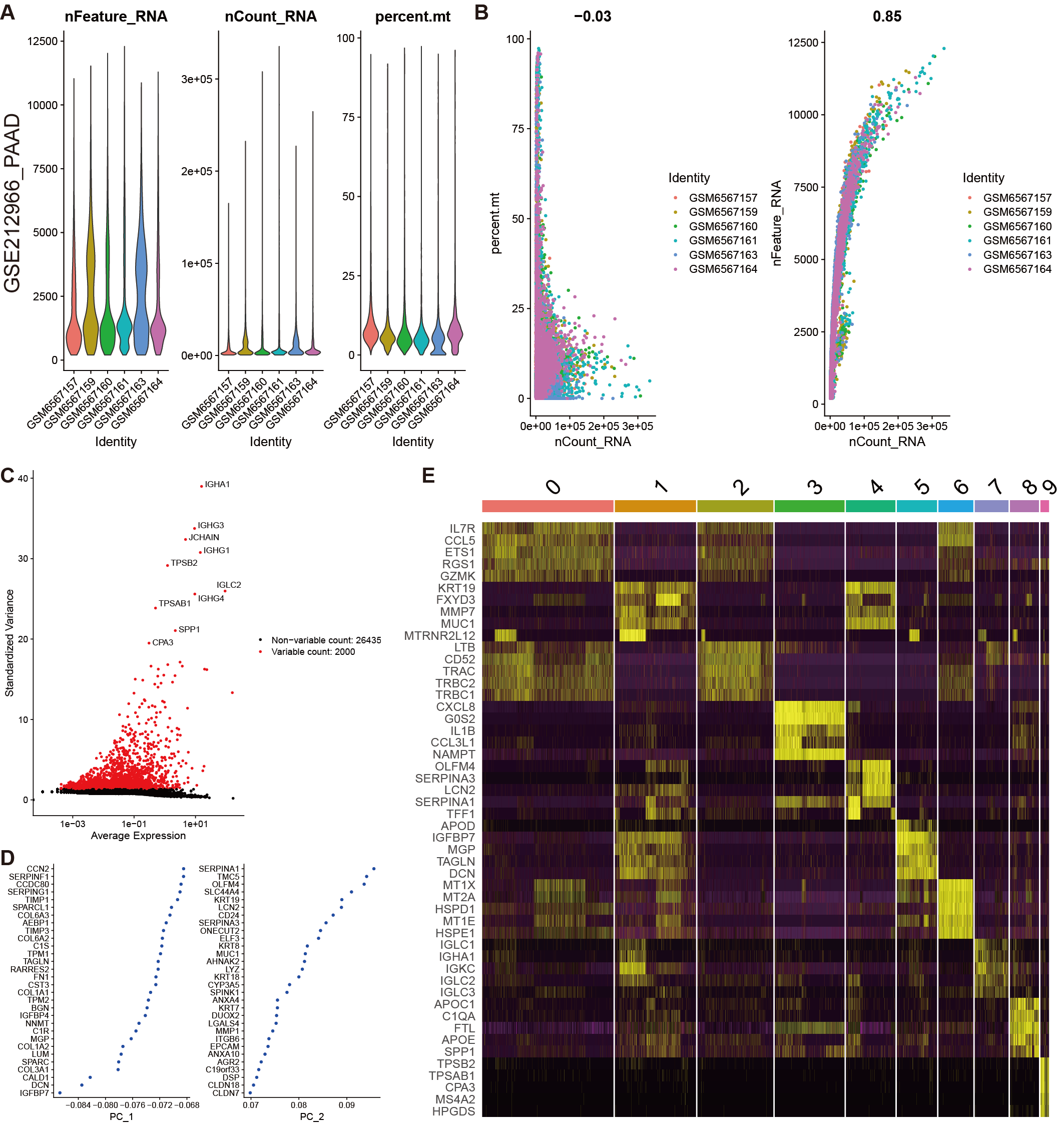

Supplement: Supplementary file 1 [file DataSheet_1.zip › Supplementary materials/Figure_S1_Quality control and key genes of 6 PAAD samples from GSE212966.tif]

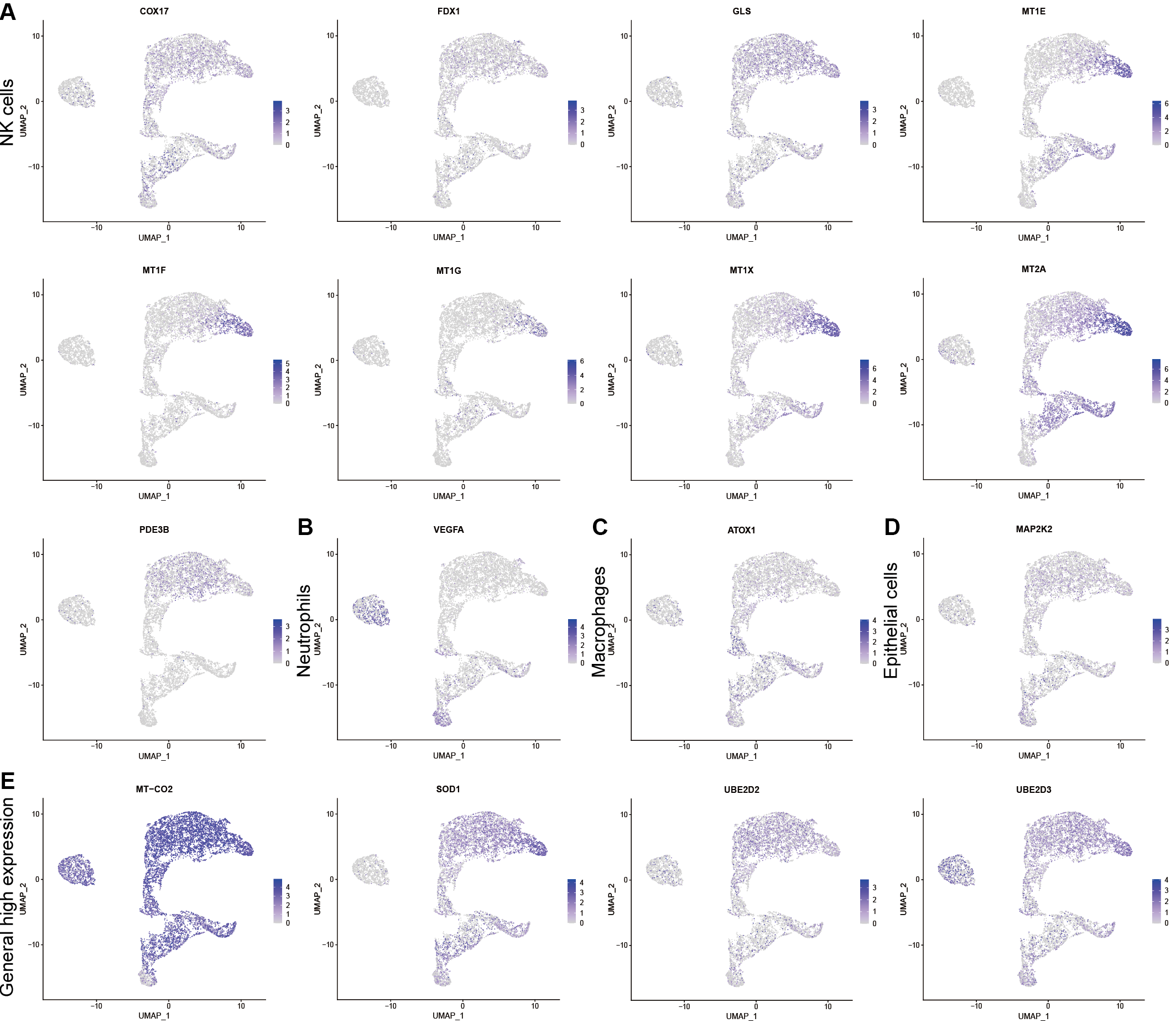

Supplement: Supplementary file 1 [file DataSheet_1.zip › Supplementary materials/Figure_S2_Significantly expressed CRGs in PAAD single-cell samples.tif]

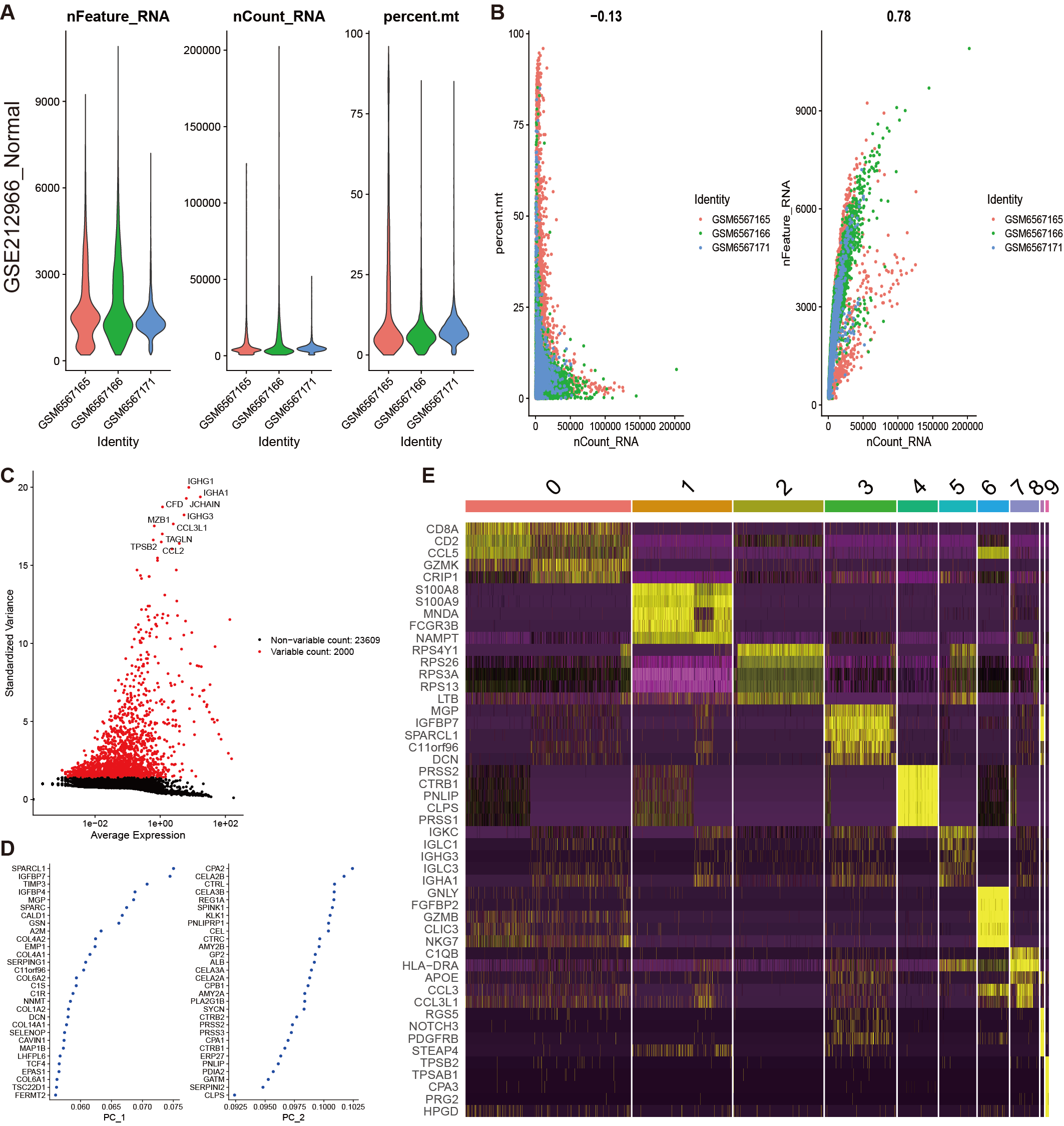

Supplement: Supplementary file 1 [file DataSheet_1.zip › Supplementary materials/Figure_S3_Quality control and key genes of 3 Normal samples from GSE212966.tif]

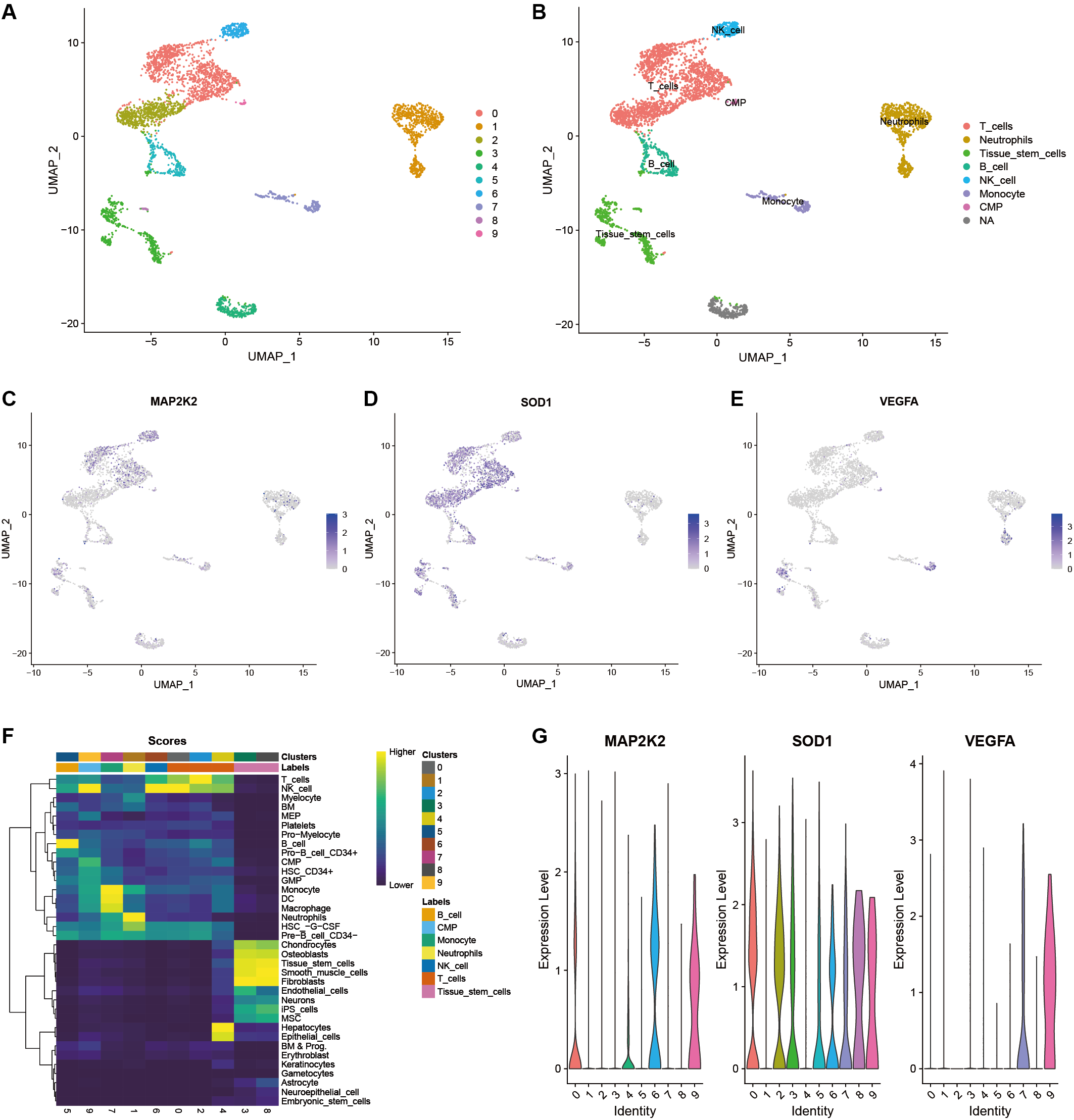

Supplement: Supplementary file 1 [file DataSheet_1.zip › Supplementary materials/Figure_S4_Expression pattern of immune-related CRGs at the GSE212966 single-cell level in normal tissues adjacent to PAAD.tif]

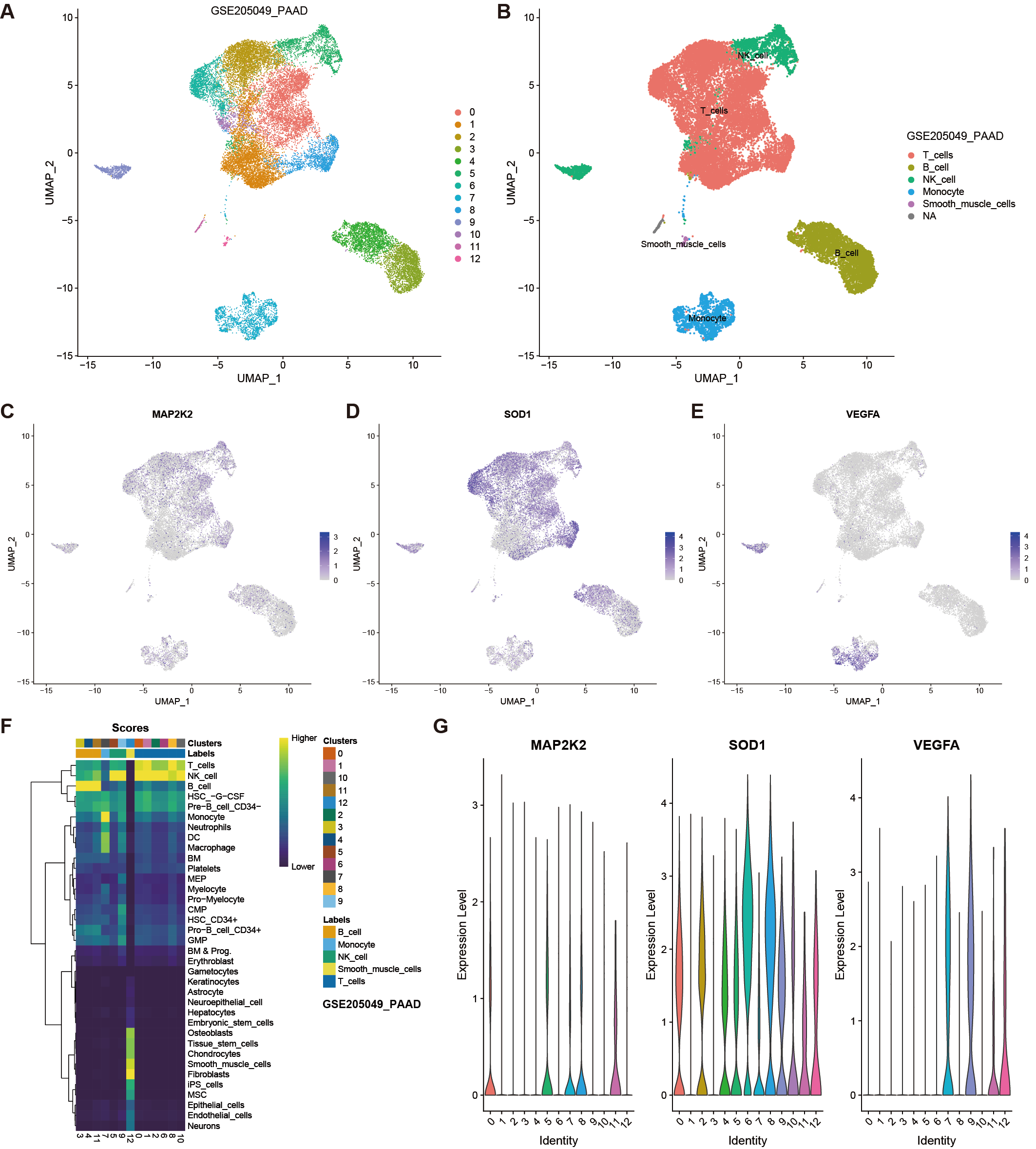

Supplement: Supplementary file 1 [file DataSheet_1.zip › Supplementary materials/Figure_S5_Expression pattern of immune-related CRGs at the level of single-cell of GSE205049 PAAD samples.tif]

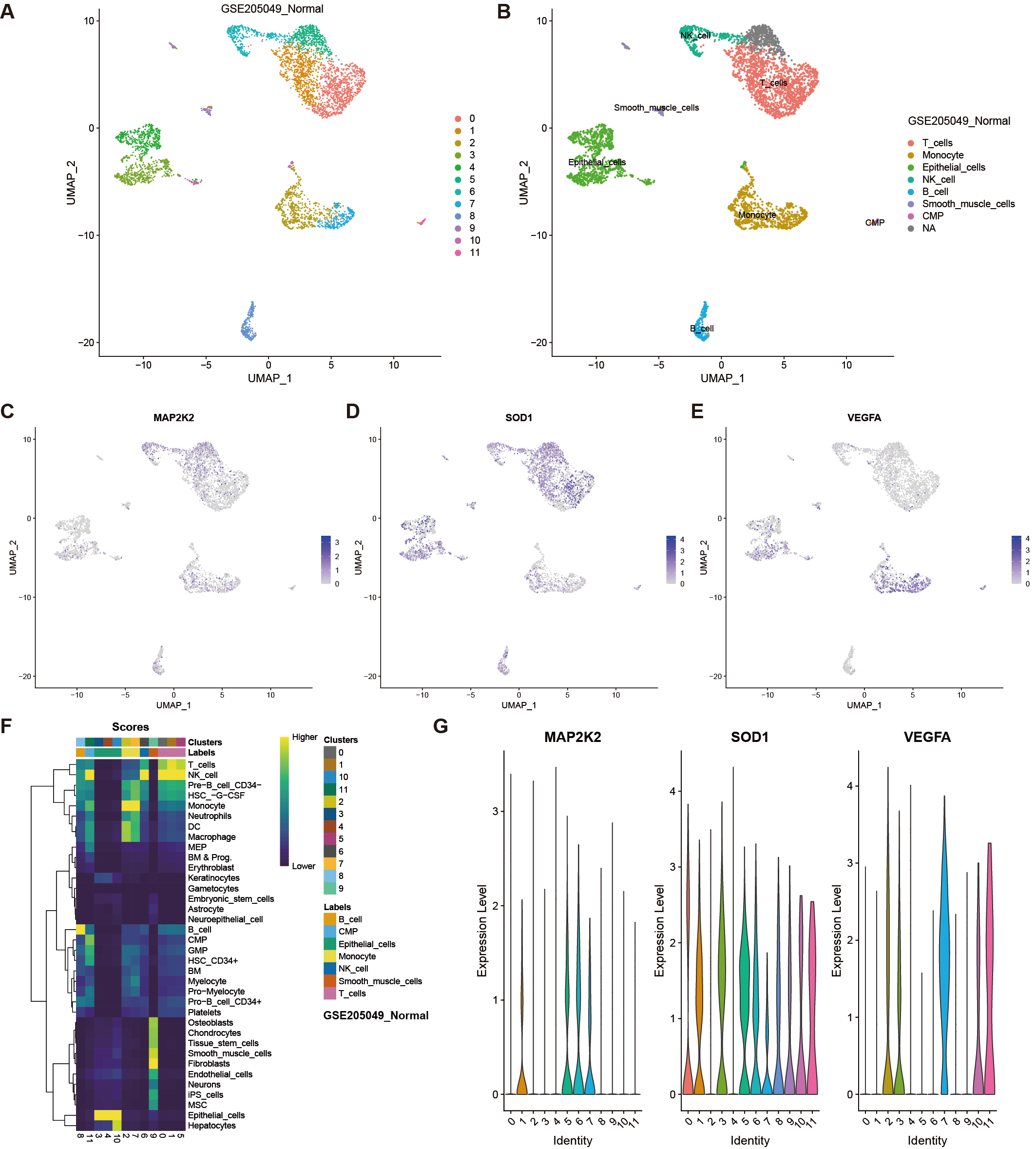

Supplement: Supplementary file 1 [file DataSheet_1.zip › Supplementary materials/Figure_S6_Expression pattern of immune-related CRGs at the level of single-cell of GSE205049 normal samples.tif]
